# Targeted Sodium Acetate Liposomes for Hepatocytes and Kupffer Cells: An Oral Dual-Targeted Therapeutic Approach for Non-Alcoholic Fatty Liver Disease Alleviation

**DOI:** 10.3390/nu17050930

**Published:** 2025-03-06

**Authors:** Yichao Hou, Xilong Gao, Jiahui Gong, Xinrui Dong, Yanling Hao, Zhengyuan Zhai, Hao Zhang, Ming Zhang, Rong Liu, Ran Wang, Liang Zhao

**Affiliations:** 1 Key Laboratory of Functional Dairy, Department of Nutrition and Health, China Agricultural University, Beijing 100193, China; b20203260995@cau.edu.cn (Y.H.); gaolong@cau.edu.cn (X.G.); haoyl@cau.edu.cn (Y.H.); liurong@cau.edu.cn (R.L.); wangran@cau.edu.cn (R.W.); 2 College of Food Science and Nutritional Engineering, China Agricultural University, Beijing 100083, China; bs20233060629@cau.edu.cn (J.G.); s20213061014@cau.edu.cn (X.D.); zhaizy@cau.edu.cn (Z.Z.); zhanghaocau@cau.edu.cn (H.Z.); 3 School of Food and Health, Beijing Technology and Business University, Beijing 100048, China; zhangming@th.btbu.edu.cn; 4 Research Center for Probiotics, China Agricultural University, Beijing 101299, China

**Keywords:** sodium acetate, non-alcoholic fatty liver disease, liposomes, hepatocyte, Kupffer cell

## Abstract

**Background/Objectives:** Sodium acetate (NaA) has demonstrated potential in improving non-alcoholic fatty liver disease (NAFLD) by targeting hepatocytes and Kupffer cells. However, its clinical application is hindered by low oral bioavailability and insufficient liver concentrations. Liposomes, with their capacity to encapsulate water-soluble drugs and be surface-modified, offer a promising solution for targeted oral drug delivery. **Methods:** We designed NaA-loaded liposomes modified with sodium cholate (SC) and mannose (MAN) (NaA@SC/MAN-LPs) to target hepatocytes and Kupffer cells. **Results:** The NaA@SC/MAN-LPs had a mean diameter of approximately 100 nm with a positive surface charge. Compared to free NaA, NaA@SC/MAN-LPs significantly extended the serum half-life from 2.85 h to 15.58 h, substantially improving in vivo bioavailability. In vivo distribution studies revealed that NaA@SC/MAN-LPs extended the acetate peak time in the liver from 15 min to 60 min and increased hepatic acetate accumulation to 3.75 times that of free NaA. In in vitro cell experiments, NaA@SC/MAN-LPs significantly reduced the lipid droplet, triglycerides (TG), and total cholesterol (TC) in a fatty acid-induced hepatocyte steatosis model and suppressed proinflammation in a lipopolysaccharide (LPS)-activated Kupffer cell inflammation model. Free NaA effectively improved hepatic lipid deposition in NAFLD mice. Furthermore, NaA@SC/MAN-LPs decreased hepatic TG, TC, and the relative area of lipid droplets by 30.44%, 15.26%, and 55.83%, compared to free NaA. Furthermore, the liposomes reduced macrophage infiltration and pro-inflammatory response. **Conclusions:** The NaA@SC/MAN-LPs demonstrated effective dual targeting effects on hepatocytes and Kupffer cells, significantly improving the pathogenesis of NAFLD, compared to free NaA. This study provides a new strategy for developing effective and safe oral drugs for NAFLD.

## 1. Introduction

Non-alcoholic fatty liver disease (NAFLD) is the leading cause of chronic liver disease globally, affecting an estimated 25% to 40% of the population [1]. The “multiple-hit” hypothesis is widely accepted as the pathogenesis underlying NAFLD, suggesting that excessive hepatic fat accumulation, insulin resistance, and other physiological changes simultaneously lead to endoplasmic stress, oxidative stress, and hepatocyte apoptosis [2]. The pathology of NAFLD is mainly manifested by lipid accumulation in hepatocytes and attenuating macrophage-mediated liver inflammation. Clinically, NAFLD progresses from simple steatosis to non-alcoholic steatohepatitis (NASH) and may advance to hepatocellular carcinoma (HCC). NAFLD is strongly associated with metabolic disease, including obesity, insulin resistance, dyslipidemia, type 2 diabetes, and cardiovascular disease [3]. Despite the disease’s prevalence, no drugs have been approved for NAFLD. The primary therapeutic strategies primarily rely on lifestyle interventions, including exercise and dietary interventions. However, the limited efficacy, prolonged duration, and challenges of long-term adherence to lifestyle interventions highlight the urgent need for effective treatments.

The gut–liver axis is a bidirectional regulatory system linking the gastrointestinal tract and liver through metabolism, immunity, and neuroendocrine interactions. Dysbiosis of gut microbiota is closely linked to NAFLD progression [4]. Gut microbiota imbalance increases gut permeability, allowing harmful microbial metabolites to enter the liver, thereby affecting hepatic metabolism and promoting the progression of NAFLD. Short-chain fatty acids (SCFAs) are common bacterial metabolites produced by gut microbiota fermentation of dietary fiber [5]. In addition to being energy substrates, SCFAs are also signaling molecules, which influences the development of NAFLD [6].

Acetate, as the most abundant SCFA, has been widely confirmed to have the effect of alleviating NAFLD. Compared to those of healthy individuals, the acetate levels in the faces of NAFLD patients are significantly reduced [7]. Additionally, the composition of the intestinal microbiota shows notable changes, primarily characterized by a reduction in SCFA-producing bacteria [8]. Oral administration of NaA also effectively alleviates lipid deposition in NAFLD rats, in whom NaA reduces liver lipid accumulation by enhancing xanthine oxidase activity and exerts protective effects on the liver [9]. Gavage acetic acid was shown to upregulate PPARα and fatty acid oxidation-related proteins through the AMPK signaling pathway, thereby inhibiting liver lipid accumulation in high-fat diet (HFD) mice [10]. In BRL-3A cells isolated from rat liver, high-dose acetate (2,4, or 8 mM) activates the AMP-activated protein kinase alpha (AMPKα) signaling pathway, leading to increased lipid oxidation and decreased lipid synthesis [11]. Additionally, acetate regulates hepatic free fatty acid receptor 2 (FFAR2) signaling, improving lipid metabolism [12]. Acetate also inhibits NAFLD progression through anti-inflammatory effects. It strengthens the intestinal barrier, reducing lipopolysaccharide (LPS) translocation to the liver and then alleviating hepatic inflammation [13]. Thus, acetate mitigates NAFLD by reducing lipid accumulation in hepatocytes and attenuating macrophage-mediated liver inflammation. However, in the liver, acetate metabolism is complex and concentration-dependent. As the raw material for lipogenesis [6], low concentrations of acetate (0.1 mM) induce lipid synthesis and promote an inflammatory response in the liver. In contrast, high concentrations of acetate (2 mM) entering hepatic macrophages suppress PPARγ regulation of UCP2 expression, decrease energy production, and then activate AMPK. In turn, AMPK inhibits the iNOS/IκBα/NFκB signaling pathway, reducing inflammatory responses in liver macrophages, which further inhibits lipid accumulation in hepatocytes [14]. Thus, effective NAFLD treatment requires sufficient acetate concentration in the liver, targeting both hepatocytes and Kupffer cells. Additionally, NaA stimulates the secretion of glucagon-like peptide 1 (GLP-1) and peptide YY (PYY) from endocrine cells through G-protein-coupled receptor transport, thereby regulating appetite and food intake, improving lipid metabolism to alleviate NAFLD [15]. Acetate inhibits gluconeogenesis and regulates insulin and glucose metabolism, thereby improving insulin resistance associated with NAFLD [5]. On the contrary, some studies report that acetate supplementation dose not significantly affect human energy consumption or fat metabolism [16]. The effective concentration of acetate in the active site is a critical factor in enhancing its therapeutic effects.

Despite its therapeutic potential, acetate’s clinical application is limited. As a low-molecular-weight compound (LMW), acetate exhibits poor pharmacokinetic properties and non-specific interactions with physiological environments. After intravenous injection, the half-life of acetate in plasma is 9.3 ± 0.6 min [17]. And after infusion into the colon, plasma acetate concentration only reaches 1.344 mM [18], which cannot achieve high therapeutic concentrations in the liver. Acetate oral supplementation is hindered by its poor palatability, poor stability, and low bioavailability [19]. Besides, high concentration of SCFAs may cause gastrointestinal damage [20]. At the same time, due to the bidirectional regulatory effect of sodium acetate on liver lipid deposition (as described earlier), a high concentration of sodium acetate uptake in the liver is essential for alleviating NAFLD. Therefore, there is an urgent need for an efficient acetate delivery system with enhanced pharmacokinetic properties, including constant and sustained liver presence, for improving NAFLD treatment. Designing targeted delivery vehicles is an important method for improving drug efficacy. Sodium acetate (NaA), as a water-soluble small molecule, is a suitable delivery carrier using liposomes. Liposomes are spherical vesicles composed of phospholipid bilayers capable of encapsulating hydrophilic and hydrophobic drugs in their aqueous core and lipid bilayer, protecting them from environmental degradation, and improving stability and bioavailability [21,22]. Liposomal properties, such as size and surface modifications, can be tailored for liver-targeted drug delivery [23].

By modifying liposomes, the intestinal permeability and liver targeting of liposomes can be improved, achieving tissue-targeted drug delivery. Bile acids specifically bind to the Na^+^-taurocholate co-transporting polypeptide (NTCP) receptor on hepatocyte surfaces and the bile acid-Na^+^-dependent bile acid transporter (ASBT) receptor on intestinal epithelial cells [24]. Sodium cholate (SC) modification enhances nanoparticle transport through the intestinal epithelium via the ASBT transporters, enabling the particles to enter hepatocytes through the NTCP transporters, which are ubiquitously expressed on the basal membrane of hepatocytes [25]. The SC-modified, liver-targeted delivery system has been successfully used to load and deliver peptides, antiviral agents, and anti-tumor drugs, enhancing liver drug absorption while reducing the associated toxic side effects [26]. In liver fibrosis mice, orally administered galangin liposomes modified by sodium bile acid significantly increase the oral bioavailability of galangin (470.12%) and exhibit enhanced hepatoprotective effects [27]. Doxorubicin- and silybin-loaded liposomes modified with DSPE-PEG-cholic acid (CA-LP-DOX/SLB) demonstrate substantial accumulation in the liver, effectively inhibiting liver tumor growth [28]. Moreover, the incorporation of bile salts into the liposomal structure prevents the disruption of the liposome by endogenous bile acids, increasing the stability and prolonging its retention time in the gastrointestinal tract [29]. The mannose (MAN) receptor, a C-type lectin receptor predominantly expressed on macrophages, recognizes glycosylated molecules containing terminal mannose. It is frequently utilized for drug delivery or immunotherapy, such as oral antigen delivery. Upon modification with mannose [30], targeted drug delivery is achieved through endocytosis mediated by mannose receptors on the macrophage surface [31]. Current liver-targeted drug delivery systems are primarily used for poorly soluble drugs or macromolecular drugs to extend absorption time and enhance oral bioavailability. And liver-targeted drug delivery systems primarily rely on single-modification. Synchronous modification of liposomes with both SC and MAN enable enhanced intestinal penetration and dual targeting of two key pathological cells involved in NAFLD. This combined approach maximizes the therapeutic effects of sodium acetate, improving the treatment of NAFLD.

Currently, liposomes have been used as delivery nanocarriers for SCFA to improve their bioavailability in vivo. M. Sahuri-Arisoylu et al. developed sodium acetate-loaded liposomes (LITA) and intraperitoneally injected them into HFD-fed C57BL/6 mice, which reduced hepatic lipid accumulation, increased thermogenesis, and decreased overall body fat [32]. Qian Chen et al. encapsulated butyrate in liposomes and created NaBu tablets [33], which effectively inhibited *F. nucleatum*-mediated chemotherapy resistance in subcutaneous colorectal tumors. However, there is a notable absence of NaA liposomes capable of simultaneously targeting both hepatocytes and Kupffer cells. Furthermore, the potential alleviation effects of dual-targeted NaA liposomes for NAFLD remain to be investigated and verified.

To overcome the challenge of low liver concentration after oral administration of sodium acetate, we designed sodium acetate-loaded liposomes modified with SC and MAN, enabling simultaneous targeting of hepatocytes and Kupffer cells. We evaluated the in vivo targeting efficiency of NaA liposomes and their potential to improve NAFLD in mice. This strategy provides a promising approach for effectively alleviating the progression of NAFLD.

## 2. Materials and Methods

### 2.1. Materials and Animals

1,2-distearoyl-sn-glycero-3-phosphoethanolamine-*N*-[amino(polyethylene glycol)-2000] (DSPE-PEG^2000^-NH_2_), 1,2-dioctadecanoyl-sn-glycero-3-phosphocholine (DSPC) were purchased from Ponsure Biotech (Shanghai, China). Cholic acid, cholesterol, 3β-[*N*-(Dimethylaminoethane)-carbamoyl]-cholesterol (DC-cholesterol), *N*-Ethyl-*N*′-(3-dimethylaminopropyl)carbodiimide (EDC), *N*-Hydroxysuccinimide (NHS), ^1–13^C-acetic acid, ^1–13^C-sodim acetate were obtained from Sigma-Aldrich (St. Louis, MO, USA). Sulfo-Cyanine7 Succinimidyl Ester triethylamine (CY7-SE triethylamine) was purchased from MedChemExpress (Princeton, NJ, USA).

Six-weeks male C57BL/6J mice were purchased from Charles River Laboratories (Wilmington, NC, USA). All animals were maintained on 12 h light/12 h darkness cycles (lights on at 06:00) and allowed access to diet and water ad libitum. All animal experimental designs were approved by the Animal Welfare and Ethics Committee of Zhongyan Zichuang (Beijing) Biotechnology Co., Ltd., (Beijing, China) (Approval No. ZYZC202402006S).

### 2.2. Synthesis of DSPE-PEG-SC and DSPE-PEG-MAN

**Figure 1:**
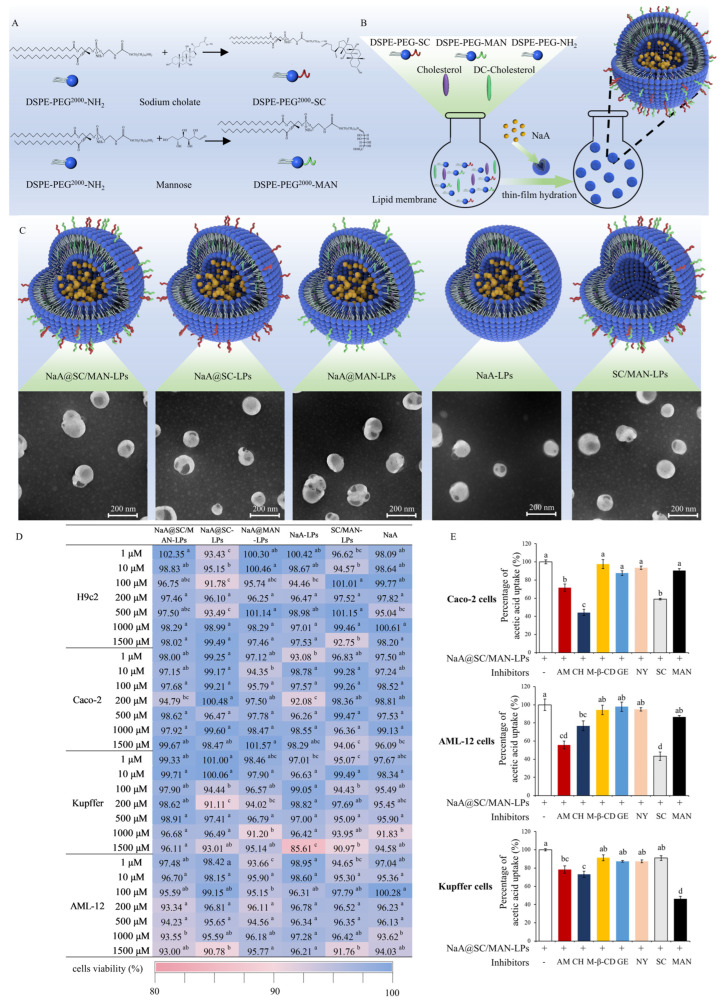
Preparation and characterization of NaA-loaded liposomes. (**A**) Schematic illustration of synthesis of DSPE-PEG^2000^-SC and DSPE-PEG^2000^-MAN. (**B**) Schematic illustration of the hepatic-targeted liposomes loaded with sodium acetate using the thin-film hydration method. (**C**) Three-dimensional diagrams and TEM images of NaA@SC/MAN-LPs, NaA@SC-LPs, NaA@MAN-LPs, NaA-LPs, and SC/MAN-LPs (scale bar = 200 nm). The red color on the surface of the liposomes indicates DSPE-PEG^2000^-SC, and the green color represents DSPE-PEG^2000^-MAN. The yellow color in the core of liposomes corresponds to NaA. (**D**) Cell viability of H9c2 cells, Caco-2 cells, Kupffer cells, and AML-12 cells after 24 h incubation in 1~1500 μM liposomes or NaA. (**E**) Percentage of NaA uptake in the presence of respective inhibitors. Data are mean ± SD. Different letters indicate significant differences (*p* < 0.05), while the same letter indicates no significant difference (*p* > 0.05). Abbreviations: NaA: sodium acetate; SC: sodium cholate; MAN: mannose; AM: amiloride hydrochloride; CH: chlorpromazine; M-β-CD: methyl-β-cyclodextrin; GE: genistrin; NY: nystatin.

Cholic acid was activated to SC using EDC and NHS. DSPE-PEG^2000^-NH_2_ was separately reacted with SC and MAN in dimethyl sulfoxide (DMSO) and triethylamine [25,34]. After dialysis and freeze-drying, the targeting materials were confirmed by differential scanning calorimeter (DSC, TA DSC250, Waters, Milford, MA, USA) and Fourier Transform Infrared Spectrometer (FTIR, Nicolet IS10, Thermo Nicolet Corporation, Waltham, MA, USA). Schematic illustrations of the synthesis are shown in Figure 1A.

### 2.3. Preparation of NaA-Loaded Liposomes

Liver-targeted NaA liposomes for oral delivery were prepared using the thin-film hydration method [35]. The formulation components included DSPC, cholesterol, DSPE-PEG^2000^-SC, DSPE-PEG^2000^-MAN, DSPE-PEG^2000^, and DC-cholesterol. These components were mixed, subjected to rotary evaporation, and hydrated with NaA stock solution. Ultrasonication was used to homogenize the mixture, followed by extrusion through a 100 nm filter membrane using extrusion apparatus (Avanti Polar Lipids, Alabaster, AL, USA). Unencapsulated NaA was removed by ultrafiltration, and the liposomes were rapidly frozen in liquid nitrogen.

The particle size, poly-dispersity indexes (PDI), and zeta-potential values of the liposomes were measured using Zetasizer Ultra (Malvern Instruments Ltd., Malvern, UK). The size and morphology were further analyzed using transmission electron microscopy (TEM, JEM-1400Flash, Tokyo, Japan). NaA content was quantitated using high-performance liquid chromatography (HPLC, Thermol Hypersil ODS-2 C18 column). Encapsulation efficiency (EE%) and drug loading (DL%) were calculated with the following formulas:

EE% = (weight of NaA in found loaded/weight of NaA input) ×100

DL% = (weight of NaA in found loaded/weight of NaA loaded liposome) ×100

Orthogonal experimental design was conducted to optimize liposome consumption. The optimal formulation and NaA stock solution was determined based on the morphology and stability in simulated body fluids. Detailed steps are provided in Supplementary Materials. Finally, the optimal formulation was established as follows: the DSPC-to-cholesterol ratio was 3:1, DSPE-PEG^2000^-SC and DSPE-PEG^2000^-MAN contents were 1% and 10%, respectively, and the NaA stock solution concentration was 40 mg/mL. Based on those parameters, the following liposome formulations were prepared: SC-MAN-dual-modified liposomes with NaA-loaded (NaA@SC/MAN-LPs), SC-modified liposomes with NaA-loaded (NaA@SC-LPs), MAN-dual-modified liposomes with NaA-loaded (NaA@MAN-LPs), unmodified liposomes with NaA-loaded (NaA-LPs), and SC-MAN-dual-modified liposomes without NaA (SC/MAN-LPs).

### 2.4. In Vitro Cytotoxicity Evaluation

H9c2 (rat myocardium), Caco-2 (human intestinal epithelial cells), Kupffer (human liver macrophages), and AML-12 (mouse hepatocyte) cells were used to evaluate the cytotoxicity of the five liposomes using the CCK-8 assay. Cells were seeded at 5 × 10^5^ cells/well in a 96-well plate and stabilized for 24 h at 37 °C in CO_2_ incubator. After stabilizing, the cells were cultured for 24 h various concentrations of NaA@SC/MAN-LPs, NaA@SC-LPs, NaA@MAN-LPs, NaA-LPs, SC/MAN-LPs, and NaA. Cell viability was evaluated using CCK-8 kit, and absorbance was measured at 450 nm using a multifunctional microplate reader (Infinite M200 Pro, TECAN, Mannedorf, Switzerland).

### 2.5. Endocytosis Pathway Activity Using Uptake Inhibitors

Cells were incubated with SC, MAN, or endocytosis inhibitors to verify the liposome endocytosis mechanism, whether it related to receptor-mediated endocytosis or micropinocytosis, clathrin-mediated, or lipid raft-mediated endocytosis. AML-12, Kupffer and Caco-2 cells were pretreated for 30 min with the following inhibitors: amiloride hydrochloride (50 μmol/L, AM), chlorpromazine (30 μmol/L, CH), methyl-β-cyclodextrin (10 mmol/L, M-β-CD), genistrin (50 μg/mL, GE), nystatin (10 μoml/L, NY), SC (5 μg/mL), and MAN (0.5 mg/mL). After the inhibitors were removed, the cells were incubated with NaA@SC/MAN-LPs for 2 h. Cellular uptake of the liposomes was determined using HPLC after cell disruption.

### 2.6. In Vivo Distribution of Fluorescent Liposomes Following Oral Intaking

**Figure 2:**
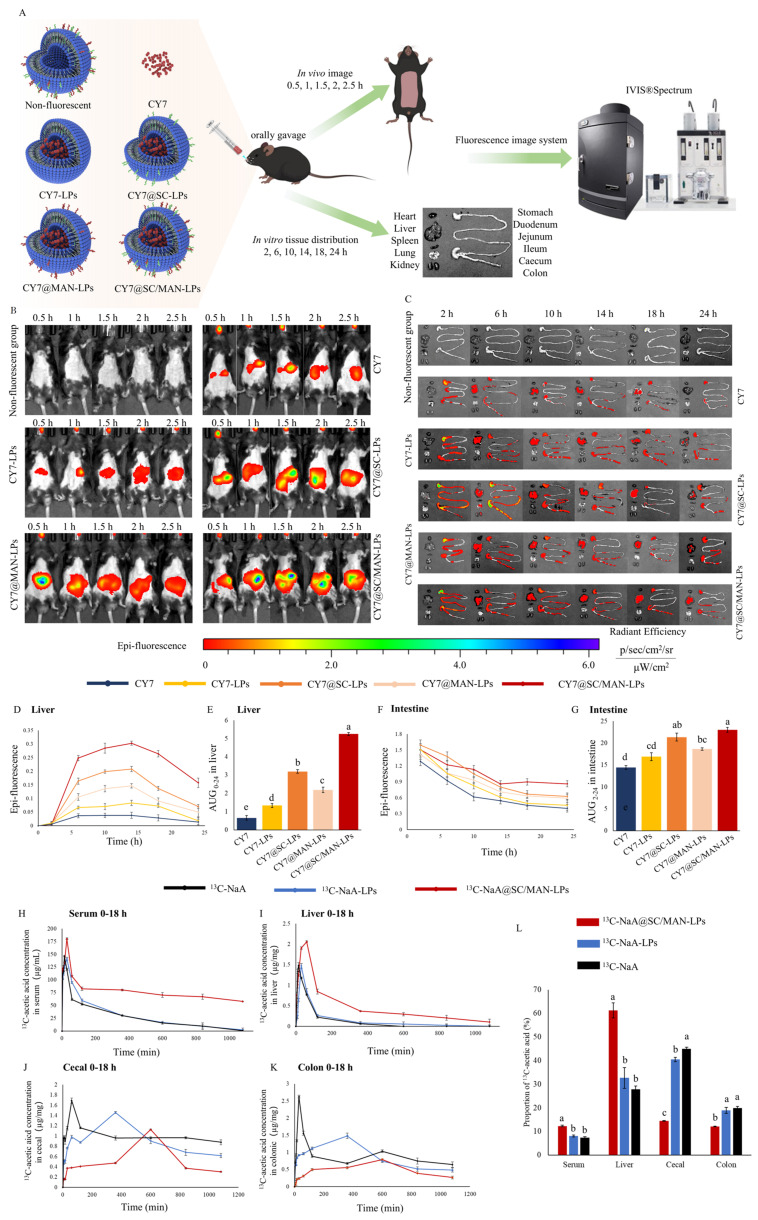
Distribution of NaA-loaded liposomes after oral administration. (**A**) Schematic diagram of in vivo fluorescence image and tissue distribution after gavage with fluorescent liposomes or CY7 fluorescent dyes; (**B**) in vivo fluorescence imaging from 0.5 to 2.5 h; (**C**) fluorescence imaging of major organs at 2, 6, 10, 14, 18, and 24 h post-oral administration; (**D**) fluorescence intensity–time curves for the liver; (**E**) AUG_0–24_ of liver fluorescence intensity-time curve in mice; (**F**) fluorescence intensity–time curve for the intestines; (**G**) AUG_2–24_ of intestines fluorescence intensity–time curve; (**H**) ^13^C-acetic acid concentration in serum between 0–18 h; (**I**) ^13^C-acetic acid concentration in liver between 0–18 h; (**J**) ^13^C-acetic acid concentration in cecum between 0–18 h; (**K**) ^13^C-acetic acid concentration in colon between 0–18 h; (**L**) proportion of ^13^C-acetic acid in serum, liver, cecum, and colon. Data are mean ± SD. Different letters indicate significant differences (*p* < 0.05), while the same letter indicates no significant difference (*p* > 0.05).

To visualize the in vivo distribution of NaA liposomes, the water-soluble, near-infrared fluorescent dye CY7-SE triethylamine was encapsulated into the liposomes. Four fluorescent liposomes were prepared: CY7@SC/MAN-LPs, CY7@SC-LPs, CY7@MAN-LPs, and CY7-LPs. Six-week-old male C57BL/6J mice were randomly divided into six groups (*n* = 3). Each group received 200 μL of either CY7@SC/MAN-LPs, CY7@SC-LPs, CY7@MAN-LPs, CY7-LPs, CY7, or non-fluorescent SC/MAN-LPs via oral gavage. Fluorescence intensity was captured at 0.5, 1, 1.5, 2, and 2.5 h post-gavage using a small animal imaging system (IVIS^®^Spectrum, Perkin Elmer, Waltham, MA, USA) under isoflurane gas anesthesia (Figure 2A). To assess fluorescent accumulation in various organs, an additional 36 male C57BL/6J mice (*n* = 6) were treated as above. Mice were euthanized at 2-, 6-, 10-, 14-, 18-, and 24-h post-gavage. Organs, including the heart, liver, spleen, lungs, kidneys, and gastrointestinal tract, were collected for fluorescence intensity measurement using IVIS^®^Spectrum.

### 2.7. Tissue Distribution of ^*13*^C-NaA-Loaded Liposomes Following Oral Intaking

NaA was replaced by ^13^C-NaA, and then, ^13^C-NaA@SC/MAN-LPs and ^13^C-NaA@LPs were prepared. Ninety C57BL/6J mice were randomly assigned to three groups (*n* = 30), receiving 200 μL of either ^13^C-NaA@SC/MAN-LPs, ^13^C-NaA-LPs, or ^13^C-NaA via oral gavage. The concentration of ^13^C-NaA in the three liposomes was 25 mg/mL. At designated time points, three mice from each group were euthanized. Blood samples were collected via cardiac puncture after eyeball extraction. The liver, cecal, and colonic contents were also collected and weighed. The contents of ^13^C-acetic acid were analyzed using triple quadrupole gas chromatography–mass spectrometry (GC-MS, HP-5ms column). ^13^C-acetic acid solutions of 1 to 1000 mg/mL were prepared, and the concentration-peak area curve was established using GC-MA. Serum, liver, or intestinal content samples were then analyzed using the internal standard method with acetic acid-d_4_. A portion of the sample was accurately weighed, homogenized, and extracted with ether. The peak area of ^13^C-acetic acid was measured by GC-MS, and the concentration of ^13^C-acetic acid in the sample was determined by referencing the standard curve.

### 2.8. In Vitro Alleviation Effect of Liposomes in NAFLD Cell Models

AML-12 cells were seeded in 6-well plate (1 × 10^6^ cells/well) and stabilized for 24 h at 37 °C in CO_2_ incubator. Cells were washed with PBS and treated with 2 mL of lipid-induction medium (0.5 mM sodium oleate and 0.25 mM sodium palmitate) to induce NAFLD. Meanwhile, AML-12 cells were treated with NaA@SC/MAN-LPs, NaA, NaA-LPs, SC/MAN-LPs, with NaA concentration standardized to 1 mM. Triglycerides (TG), total cholesterol (TC), and cell protein concentration were quantified. Lipid accumulation was visualized using Oil Red O staining

Kupffer cells were induced with 20 ng/mL LPS to create an NAFLD inflammatory model. Cells were grouped into control, NAFLD inflammation model, NaA@SC/MAN-LPs, NaA, NaA-LPs, and SC/MAN-LPs treatment groups (NaA concentration standardized to 1 mM).

### 2.9. In Vivo Alleviation Effect of Liposomes in NAFLD Model Mice

Forty-eight C57BL/6J mice (weight 18 ± 1.0 g) were acclimatized for one week and then randomly divided into six groups for 10-week gavage experiment. The specific groups were as follows: (i) control group: normal diet (D12450J, Research Diets) with PBS; (ii) NAFLD model group: HFD (D12492, Research Diets) diet with PBS; (iii) HFD diet with NaA@SC/MAN-LPs; (iv) HFD diet with NaA; (v) HFD diet with NaA-LPs; (vi) HFD diet with SC/MAN-LPs. NaA concentration was standardized to 15 mg/mL, with gavage every other day.

Body weight was recorded weekly at fixed times. Oral glucose tolerance tests (OGTT) were performed one day before the mice were sacrificed. Post-intervention, mice were euthanized, and tissues (liver, intestine, epididymal fat, perirenal fat, and inguinal fat) were collected, weighed, and analyzed. H&E staining and Oil Red O staining were performed on liver and fat samples. Liver F4/80 immunohistochemical staining was performed by Servicebio Technology Co., Ltd. (Wuhan, China). All slices were imaged using digital pathology slide scanner and analyzer (VENTANA, Camperdown, Australia, DP200) and analyzed using ImageJ (v1.53e).

Serum biochemical analyses were analyzed with an automatic biochemical analyzer (Mindray Bio-Medical Electronics, Mahwah, NJ, USA, BS-430), including TG, TC, high-density lipoprotein cholesterol (HDL-C), low-density lipoprotein cholesterol (LDL-C), alanine aminotransferase (ALT), and aspartate aminotransferase (AST). Liver TG, TC were quantified using kits (A110-1-1, A111-1-1, A003-1, A001-3, Nanjing Jiancheng Bioengineering Institute).

### 2.10. qRT-PCR

Total RNA was extracted using the SteadyPure rapid RNA extraction kit (Accurate Biology, Hangzhou, China, AG21023) and reverse-transcribed using an Evo M-MLV RT Mix Kit for qPCR (Accurate Biology, AG11728). qRT-PCR was performed using SYBR^®^ Green Premix Pro Taq HS qPCR Kit (Accurate Biology, AG11701) on the Light-Cycler 480 (Roche Diagnostics) and analyzed by the LightCycler^®^96 1.1.0.1315 (Roche Diagnostics, Sydney, Australia). 2^−ΔΔCt^ was used to calculate gene expression, which was normalized to Gapdh expression for a relative level (Primers listed in Table S3).

### 2.11. Western Blot

Total protein was extracted using a protein assay kit (23225, Sigma, Macquarie Park, Australia). Membranes were incubated with primary antibodies against NF-κB p65, *P*p65, AMPK, and *p*AMPK (8242, 3033, 5831, 2535, Cell Signaling Technology, Danvers, MA, USA). Goat anti-rabbit horseradish peroxidase-conjugated secondary antibody (ZB-2301, ZSGB-BIO, Beijing, China) was used, and signals were detected using a Super ECL Plus Enhanced Chemiluminescence Kit (P1030, Applygen Technologies Inc., Beijing, China) and detected by Imager 600 (Amersham, Switzerland). The band intensities were analyzed using Image Lab 6.1.

### 2.12. Statistical Analyses

Experiment data were presented as means ± standard deviation (SD). The *t*-test method was used to perform significance analysis between two groups of data. Variables were tested for normality using a Shapiro–Wilk test. Statistical differences were analyzed by Kruskal–Wallis test and the Duncan to adjust for multiple comparison. Statistical comparisons were performed by SPSS 26.0 (Norman Nie, Chicago, IL, USA). Probability (*p*) value less than 0.05 was regarded as statistically significant.

## 3. Results

### 3.1. Synthesis of DSPE-PEG^*2000*^-SC and DSPE-PEG^*2000*^-MAN

SC and MAN were conjugated to DSPE-PEG^2000^-NH_2_, as illustrated schematically in Figure 1A. The synthesized products were monitored by DSC and FTIR, with results shown in Figure S1. DSC analysis revealed notable changes in thermal properties. The thermal curve of DSPE-PEG^2000^-SC showed a reduction in the characteristic melting temperature (from 53.11 °C to 47.0 °C) and the appearance of a new high-temperature endothermic peak at 198.96 °C, suggesting that SC altered the PEG chain arrangement and crystalline structure. Similarly, for DSPE-PEG-MAN, the melting temperature of the PEG chain endothermic peak decreased to 46.1 °C, and the characteristic endothermic peak of MAN disappeared, further confirming conjugation. FTIR spectra supported these findings. For DSPE-PEG^2000^-SC, a characteristic peak at 1551 cm^−1^ was observed, corresponding to the stretching vibration of a carboxylate group (C=O). Additionally, a hydroxyl group (-OH) stretching vibration appeared at 3425 cm^−1^. Peaks between 2850 and 2917 cm^−1^ were associated with C-H bond stretching in the stearoyl chains of DSPE-PEG. The FTIR spectrum of DSPE-PEG^2000^-MAN showed peaks at 3428 cm^−1^, 2917 cm^−1^, and 1739 cm^−1^, indicating the presence of hydroxyl groups, stearoyl alkyl chains (C-H group), and aldehyde C=O group, respectively. These results collectively confirm the successfully conjugation of SC and MAN to DSPE-PEG^2000^-NH_2_.

### 3.2. Design and Preparation of NaA Liposomes

Liver-targeted, NaA-loaded liposomes for oral delivery were prepared using the thin-film hydration method, as depicted in Figure 1B. The ratios of DSPC to cholesterol, DSPE-PEG^2000^-SC, and DSPE-PEG^2000^-MAN were optimized using orthogonal experimental design, while the concentration of NaA stock solution was further optimized. Detailed optimization results are provided in the Supplementary Materials (Figures S2 and S3). The final optimal formulation consisted of 40.5% DSPC, 13.5% cholesterol, 1% DSPE-PEG^2000^-SC, 10% DSPE-PEG^2000^-MAN, 5% DSPE-PEG^2000^, and 30% DC-cholesterol, with a NaA stock solution concentration of 40 mg/mL.

### 3.3. Characterization of NaA Liposomes

Based on liposome composition, five distinct liposome types were synthesized: NaA@SC/MAN-LPs, NaA@SC-LPs, NaA@MAN-LPs, NaA-LPs, and SC/MAN-LPs. Their particle size, PDI, zeta potential, EE%, and DL% were measured, as shown in Table 1. All liposomes displayed particle sizes close to 100 nm, a PDI < 0.25, and a positive zeta potential. For NaA@SC/MAN-LPs, NaA@SC-LPs, NaA@MAN-LPs, and NaA@LPs, EE% ranged from 76.6% to 81.57%, while DL% ranged from 33.16% to 37.94%, confirming high encapsulation and loading efficiency for NaA. TEM images (Figure 1C) further verified the morphology of the liposomes. They exhibited a spherical shape, consistent with particle size measurements, and demonstrated a homogeneous distribution without aggregation.

### 3.4. Cell Cytotoxicity Test of NaA Liposomes

The cytotoxicity of NaA@SC/MAN-LPs, NaA@SC-LPs, NaA@MAN-LPs, NaA-LPs, SC/MAN-LPs, and NaA was evaluated using the CCK-8 assay in H9c2, Caco-2, Kupffer, and AML-12 cells. NaA concentrations ranged from 1 to 1500 μM. As shown in Figure 1D, after incubating the five liposomes with NaA concentrations ranging from 1 to 1500 μM for 24 h, cell viability in H9c2, Caco-2, Kupffer, and AML-12 cells remained above 85%. These results indicate that the unmodified liposomes, as well as the SC- and MAN-modified NaA liposomes, are nontoxic.

### 3.5. Endocytosis Pathway Activities Using Uptake Inhibitors

To investigate the uptake mechanisms of NaA@SC/MAN-LPs, inhibitors were applied as shown in Figure 1E. SC significantly inhibited the uptake of NaA@SC/MAN-LPs by AML-12 cells and Caco-2 cells (*p* < 0.05), while MAN significantly inhibited uptake by Kupffer cells (*p* < 0.05). This suggests that NaA facilitates NaA@SC/MAN-LPs transport through ASBT and NTCP receptors in hepatocyte and intestinal cells. Similarly, MAN receptor-mediated transport of NaA@SC/MAN-LPs was observed in Kupffer cells. Further studies with inhibitors revealed that amiloride hydrochloride and chlorpromazine significantly reduced the uptake of NaA@SC/MAN-LPs in AML-12 cells, Kupffer cells, and Caco-2 cells (*p* < 0.05). Conversely, methyly-β-cyclodextrin, genistrin, and nystatin showed no significant effect on cellular uptake. Amiloride hydrochloride inhibits micropinocytosis, while chlorpromazine could prevent clathrin-mediated endocytosis by disrupting the assembly of the clathrin adaptor protein at the cell surface. Therefore, these findings indicated that the uptake of NaA@SC/MAN-LPs involved both macropinocytosis and clathrin-mediated endocytosis in AML-12, Kupffer, and Caco-2 cells.

### 3.6. Biodistribution and In Vivo Imaging of Fluorescent Liposomes

The in vivo fluorescence imaging after oral administration of non-fluorescent liposomes, CY7, CY7-LPs, CY7@SC-LPs, CY7@MAN-LPs, and CY7@SC/MAN-LPs are shown in Figure 2A,B. Mice receiving CY7 showed low fluorescence intensity throughout the 0 to 2.5-h period. In contrast, those administered CY7@SC/MAN-LPs, CY7@SC-LPs, CY7@MAN-LPs, and CY7-LPs showed fluorescence intensity accumulation in the abdominal region and prolonged fluorescence retention time. The accumulation of the modified liposomes in the abdominal region was ranked as follows: CY7@SC/MAN-LPs > CY7@SC-LPs > CY7@MAN-LPs. These results indicate that the SC and MAN dual-labeled liposomes significantly increase the in vivo fluorescence intensity and retention time of CY7.

To further investigate the distribution of these modified liposomes in various organs, the heart, liver, spleen, lungs, kidneys, and gastrointestinal tract were harvested, as shown in Figure 2C–G. In the liver, the fluorescence intensity curves demonstrated an initial increase, followed by a decline. The peak fluorescence intensity in the liver at 14 h was recorded as follows: 0.303, 0.208, 0.147, 0.084, and 0.038 [(p/sec/cm^2^/sr)/μM/cm^2^] for the CY7@SC/MAN-LPs, CY7@SC-LPs, CY7@MAN-LPs, CY7-LPs, and CY7 groups, respectively. The accumulated fluorescence intensity of CY7@SC/MAN-LPs in the liver was 1.64, 2.40, 3.93, and 7.97 times higher than that of CY7@SC-LPs, CY7@MAN-LPs, CY7-LPs, and CY7 dye, respectively. These findings indicate that SC and MAN dual-modified liposomes significantly increase the liver accumulation of fluorescent intensity after oral administration. Additionally, the fluorescence intensity in the intestines was measured, with corresponding intensity–time curves, shown in Figure 2F,G. The fluorescence intensity gradually decreased from 2 to 24 h. Notably, the fluorescence intensity of CY7@SC/MAN-LPs in the intestines was significantly higher than that of CY7@MAN-LPs, CY7-LPs, and CY7. No significant differences were observed between CY7@SC-LPs and CY7@SC/MAN-LPs. This suggests that SC and MAN dual-modification delayed the intestinal efflux of liposomes, thereby increasing the accumulation of fluorescence in the intestine.

In summary, SC and MAN dual-labeled fluorescent liposomes significantly enhance both intestinal permeability and liver targeting. These findings highlight the effectiveness of the dual-modified liposomes in prolonging retention time and enhancing liver accumulation, thereby providing a more efficient delivery system for NaA.

### 3.7. Tissue Distribution of ^*13*^C-NaA Liposomes

C57BL/6J mice were orally gavaged with ^13^C-NaA@SC/MAN-LPs, ^13^C-NaA-LPs, and ^13^C-NaA. Serum, liver, cecum, and colon samples were collected, and the concentration of ^13^C-acetic acid was measured using triple quadrupole GC-MS. The concentration–time curves were plotted as shown in Figure 2H–L.

In serum, the concentration of ^13^C-acetate acid rapidly increased, with the peak value of ^13^C-acetic acid of ^13^C-NaA group mice observed at 15 min. In contrast, the peak serum ^13^C-acetic acid concentrations in ^13^C-NaA@SC/MAN-LPs and ^13^C-NaA-LPs were delayed, occurring at 30 min. Moreover, the peak serum ^13^C-acetic acid concentration in the ^13^C-NaA@SC/MAN-LPs group (180.31 μg/mL) was higher compared to the ^13^C-NaA-LPs (141.31 μg/mL) and ^13^C-NaA (146.07 μg/mL) groups. After reaching the peak, the serum concentration of ^13^C-acetic acid in the ^13^C-NaA@SC/MAN-LPs group decreased rapidly within 2 h. After 18 h, the concentration remained at 57.75 μg/mL, significantly higher than in the ^13^C-NaA-LPs and^13^C-NaA groups. The metabolic kinetic parameters of ^13^C-acetic acid liposomes in serum are provided in Table S7. Compared to the ^13^C-NaA-LPs or ^13^C-NaA groups, the peak serum ^13^C-acetic acid concentration and AUG_0–18_ in the ^13^C-NaA@SC/MAN-LPs group was significantly increased. Additionally, the half-life of ^13^C-acetic acid in the ^13^C-NaA@SC/MAN-LPs group was extended from 3.39 h and 2.85 h to 15.58 h. These findings demonstrate that encapsulating NaA in dual-modified liposomes effectively increases the serum acetate concentration following oral administration, prolongs the retention time, and achieves a sustained release effect in vivo.

In Figure 2I, the liver ^13^C-acetic acid concentration in three groups rapidly increased, reached its peak, and then quickly decreased. In the ^13^C-NaA or ^13^C-NaA-LPs groups, the peak concentrations of ^13^C-acetic acid were 1.48 and 1.47 μg/mL, occurring at 15 min and 30 min, respectively. However, the peak concentration in the ^13^C-NaA@SC/MAN-LPs group reached 2.06 μg/mL at 60 min. Ten hours post-gavage, the liver concentration of ^13^C-acetic acid in the ^13^C-NaA and ^13^C-NaA-LPs groups had returned to baseline, while the concentration in the ^13^C-NaA@SC/MAN-LPs group remained at 0.29 μg/mL, indicating prolonged retention of ^13^C-NaA in the liver. This suggests that encapsulating NaA in dual-modified liposomes effectively enhances hepatic bioavailability and extends its duration in the liver. As shown in Figure 2J,K, the peak concentration of ^13^C-acetic acid in cecal and colonic contents in the ^13^C-NaA@SC/MAN-LPs group occurred at 10 h, longer than the ^13^C-NaA-LPs and ^13^C-NaA groups. Meanwhile, the peak concentrations of ^13^C-acetic acid were higher in the ^13^C-NaA group compared to the ^13^C-NaA@SC/MAN-LPs and ^13^C-NaA-LPs groups. Figure 2L shows the proportion of ^13^C-acetic acid absorbed by serum, liver, cecal, and colonic content. The ^13^C-acetic acid content in the serum of the ^13^C-NaA@SC/MAN-LPs group was significantly higher than that of the ^13^C-NaA-LPs and ^13^C-NaA groups. This trend was consistent in the liver, where the ^13^C-acetic acid accumulation proportion in the ^13^C-NaA@SC/MAN-LPs group was 1.8 and 2.2 times higher than the ^13^C-NaA-LPs and ^13^C-NaA groups. Due to the lack of liver-targeting function, the ^13^C-acetic acid proportion in the cecum and colon was higher in the ^13^C-NaA-LPs group compared to the ^13^C-NaA@SC/MAN-LPs group.

These results demonstrate that the SC and MAN dual-labeled NaA liposomes successfully achieved liver-targeted enrichment. Moreover, the NaA@SC/MAN-LPs exhibited significantly higher peak concentrations and prolonged retention times in the liver compared to SC or MAN single-labeled liposomes, unlabeled liposomes, or NaA alone. This approach effectively addresses the challenges of liver targeting and insufficient effective concentrations of NaA.

### 3.8. In Vitro Inhibition Efficacy on Lipid Accumulation in NAFLD Cells

**Figure 3:**
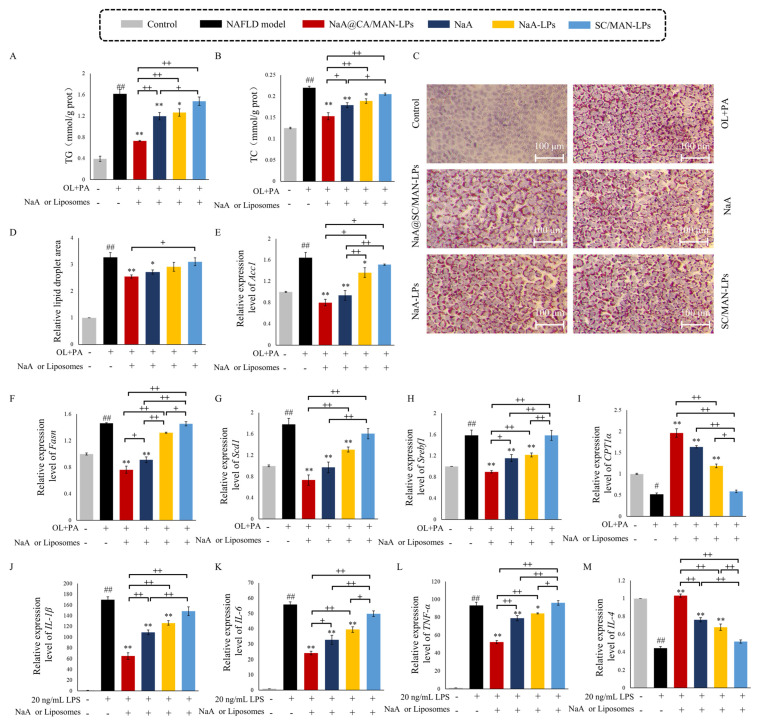
NaA liposomes or NaA solution alleviated lipid accumulation in NAFLD cell model. (**A**) Effects on cellular TG content; (**B**) effects on cellular TC content; (**C**) Oil Red O staining for different groups; (**D**) effects on the relative lipid droplet area; (**E**–**I**) lipid accumulation and fatty acid oxidation-related gene expression (*Acc1*, *Fasn*, *Scd1*, *Srebf1*, *CPT1*α); (**J**–**M**) inflammatory-related genes in LPS induced NAFLD cell model (IL-1β, IL-6, TNF-α, IL-4). Data are mean ± SD. ^#^ *p* < 0.05, ^##^ *p* < 0.01, versus control group; * *p* < 0.05, ** *p* < 0.01, versus NAFLD group; + indicates a significance between treatment groups, ^+^ *p* < 0.05, ^++^ *p* < 0.01. OL: sodium oleate; PA: sodium palmitate; LPS: lipopolysaccharide.

To construct a NAFLD lipid accumulation model, AML-12 hepatocyte was treated with 0.5 mM sodium oleate (OL) and 0.25 mM sodium palmitate (PA) for 24 h. Following induction, AML-12 cells were incubated with 1 mM NaA@SC/MAN-LPs, NaA solution, NaA-LPs, or SC/MAN-LPs. As shown in Figure 3A–D, compared to the control group, the NAFLD model group exhibited significantly increased TG and TC, as well as a larger relative area of Oil Red O-stained lipid droplets, confirming successful model establishment. Treatment with NaA@SC/MAN-LPs and NaA solution significantly reduced intracellular TG, TC, and lipid droplet accumulation compared to the NAFLD model group. Similarly, NaA-LPs demonstrated a significant decrease in TG and TC levels. However, the reduction in lipid droplet area was not significant. Incubation with SC/MAN-LPs resulted in insignificant changes, confirming that empty liposome does not aggravate lipide deposition. Among the groups, NaA@SC/MAN-LPs had a significantly stronger effect on reducing intracellular TG and TC levels than NaA solution alone. These results demonstrate that NaA-loaded SC and MAN-modified liposomes effectively alleviate lipid deposition in NAFLD cells, offering superior therapeutic potential compared to NaA alone.

To investigate the effects of NaA-loaded liposome treatment on lipid synthesis and storage in AML-12, RT-qPCR was used to assess the expression of key lipid synthesis and regulatory genes (Figure 3E–H). These genes included acetyl-CoA carboxylase 1 (*Acc1*), fatty acid synthase (*Fasn*), stearoyl-CoA desaturase 1 (*Scd1*), and sterol regulatory element-binding transcription factor 1 (*Srebf1*). After 24 h of induction with 0.5 mM OL and 0.25 mM PA, the AML-12 cells showed significant upregulation of *Acc1*, *Fasn*, *Scd1*, and *Srebf1* compared to the control group. Treatment with NaA@SC/MAN-LPs and NaA significantly reduced these gene levels relative to the NAFLD group. NaA-LPs also alleviated the upregulation of *Acc1*, *Scd1*, and *Srebf1* genes in NAFLD cells. Conversely, SC/MAN-LPs showed no significant impact on the expression of lipid synthesis genes. Notably, *Fasn* and *Srebf1* levels were significantly lower in the NaA@SC/MAN-LPs group compared to the NaA group, confirming the enhanced efficacy of NaA-loaded dual-modified liposomes in suppressing lipid synthesis-related gene expression. To further explore fatty acid oxidation, carnitine palmitoyltransferase 1α (*CPT1*α) expression was assessed, as shown in Figure 3I. In the NAFLD model group, *CPT1*α expression was significantly decreased compared to the control group. Treatment with NaA@SC/MAN-LPs, NaA, and NaA-LPs significantly upregulated *CPT1*α expression, with NaA@SC/MAN-LPs demonstrating a significantly stronger effect than unmodified NaA liposomes. These findings highlight the superior efficacy of NaA@SC/MAN-LPs in reducing lipid synthesis and promoting fatty acid oxidation in NAFLD cells compared to NaA solution.

To assess the effects of NaA liposomes on macrophage inflammatory activation, a key pathogenic factor in NAFLD, we induced inflammation in mouse liver macrophages (Kupffer cells) using 20 ng/mL LPS. The expression of inflammation-related genes was assessed following treatment with NaA@SC/MAN-LPs, NaA, NaA-LPs, and SC/MAN-LPs (Figure 3J–M). LPS treatment significantly upregulated pro-inflammatory cytokines (IL-1β, IL-6, and TNF-α) and downregulated anti-inflammatory cytokine IL-4 in Kupffer cells, indicating a robust inflammatory response in the model group. Treatment with NaA@SC/MAN-LPs, NaA-LPs, or NaA significantly reduced the expression of IL-1β, IL-6, and TNF-α, while significantly increasing IL-4 expression, demonstrating their efficacy in alleviating inflammatory activation in Kupffer cells. CA/MAN-LPs showed no significant impact on either pro-inflammatory or anti-inflammatory cytokines, indicating minimal influence on the inflammatory state of Kupffer cells. In treatment groups, NaA@SC/MAN-LPs exerted significantly stronger suppression of IL-1β, IL-6, and TNF-α mRNA expression, alongside greater enhancement of IL-4 expression, compared to NaA-LPs and NaA treatments. These results demonstrate that NaA@SC/MAN-LPs effectively mitigate the inflammatory response in Kupffer cells, with superior efficacy over both sodium acetate and unlabeled NaA liposome treatments.

### 3.9. In Vivo Alleviation Efficacy on Metabolic Abnormalities in NAFLD Mice

**Figure 4:**
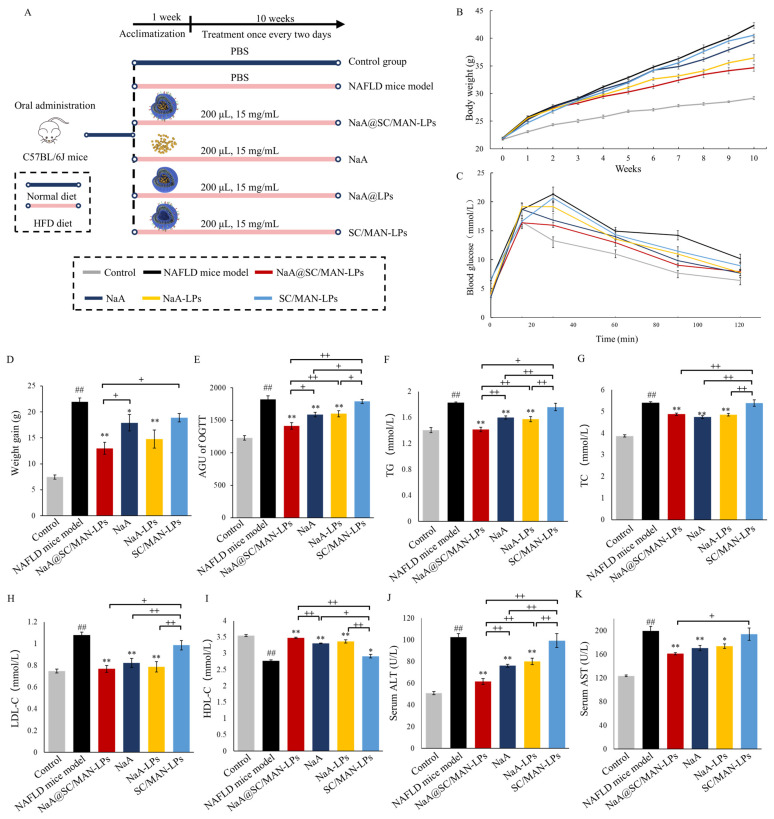
Effects of NaA liposomes or NaA solution after oral administration on metabolic abnormalities and serum hepatic function indicators of NAFLD mice. (**A**) Schematic diagram of the animal grouping and treatment; (**B**) body weight change; (**C**) weight gain; (**D**) OGTT curve; (**E**) AUG of OGTT; (**F**) TG content; (**G**) TC content; (**H**) LDL-C content; (**I**) HDL-C content; (**J**) serum ALT; (**K**) serum AST. Data are mean ± SD. *^##^ p* < 0.01, versus control group; * *p* < 0.05, ** *p* < 0.01, versus NAFLD group; ^+^ indicates a significance between treatment groups, ^+^ *p* < 0.05, ^++^ *p* < 0.01.

The animal grouping and treatment design are illustrated in Figure 4A. C57BL/6J mice were fed an HFD containing 60% fat for 10 weeks, resulting in a significant weight increase in the NAFLD mouse model group, from 21.97 g to 42.33 g. At the end of the intervention, the body weight of the NAFLD model mice was 1.45 times higher than that of the control group. The weight gain in the NAFLD model group (21.94 g) was 2.94 times that of the control group (7.47 g), with a statistically significant difference (Figure 4B,D). These results indicate that a 10-week HFD successfully induced weight gain, thereby establishing the NAFLD model.

After 10 weeks of intervention with NaA@SC/MAN-LPs, NaA-LPs, or NaA solution, the weight of the NAFLD mice decreased by 18.08%, 13.88%, and 6.45%, respectively. These groups exhibited significantly less weight gain than the NAFLD model group, indicating that both NaA and NaA liposomes effectively alleviated weight gain in NAFLD mice. In contrast, treatment with the SC/MAN-LPs resulted in a modest weight decrease of only 1.76 g after 10 weeks, with no significant decrease compared to the NAFLD model group, suggesting that SC/MAN-LPs did not impact the body weight of the NAFLD model mice. Further analysis showed that NaA@SC/MAN-LPs resulted in significantly lower body weight compared to both NaA-LPs and NaA, indicating that the SC and MAN dual-labeled liposome was more effective in alleviating weight gain in NAFLD mice than NaA treatment alone.

The results of the effects of NaA liposomes on OGTT are shown in Figure 4C,E. The NAFLD model mice exhibited significantly elevated blood glucose levels. Compared to the NAFLD group, the AUG_OGTT_ was significantly reduced in the groups treated with NaA@SC/MAN-LPs, NaA, and NaA-LPs (*p* < 0.01), indicating a significant restoration in glucose tolerance in these groups. Conversely, treatment with SC/MAN-LPs resulted in a decrease, but the difference was not significant. In treatment groups, NaA@SC/MAN-LPs were more effective than both NaA-LPs and NaA in reducing the AUG. These results suggest that NaA, in both its free form and encapsulated in liposomes, improved glucose tolerance in NAFLD mice, with the SC and MAN dual-labeled NaA liposomes demonstrating the most pronounced effect.

The serum levels of TG, TC, LDL-C, and HDL-C were investigated to explore the effect of NaA liposomes on lipid metabolism in NAFLD mice, as shown in Figure 4F–I. Treatment with NaA@SC/MAN-LPs, NaA, and NaA-LPs resulted in significant reductions in serum TG, TC, and LDL-C levels, alongside significant increases in HDL-C levels (*p* < 0.01) in NAFLD mice. However, treatment with SC/MAN-LPs did not significantly alter levels of TG, TC, or LDL-C, which suggests that the SC/MAN-LPs did not exacerbate lipid metabolism in NAFLD mice. In NaA treatment groups, NaA@SC/MAN-LPs led to a significantly lower serum TG level and a higher HDL-C level compared to NaA, which indicates that the SC and MAN dual-labeled NaA liposomes have a superior effect in alleviating lipid abnormalities in NAFLD mice compared to NaA treatment alone. Similar to serum lipid metabolism results, NaA liposomes had the same improvement trend on liver function parameters (Figure 4J,K). NaA@SC/MAN-LPs, NaA, and NaA-LPs led to a significant decrease in both ALT and AST levels, compared to the NAFLD model group. Furthermore, the NaA@SC/MAN-LPs group had significantly lower ALT levels than the SC/MAN-LPs and NaA groups. These results suggest that the SC and MAN dual-labeled NaA liposomes effectively mitigate liver damage in NAFLD mice by lowering serum ALT and AST levels, demonstrating superior efficacy compared to NaA treatment alone.

### 3.10. In Vivo Inhibition on Lipid Accumulation in NAFLD Mice

**Figure 5:**
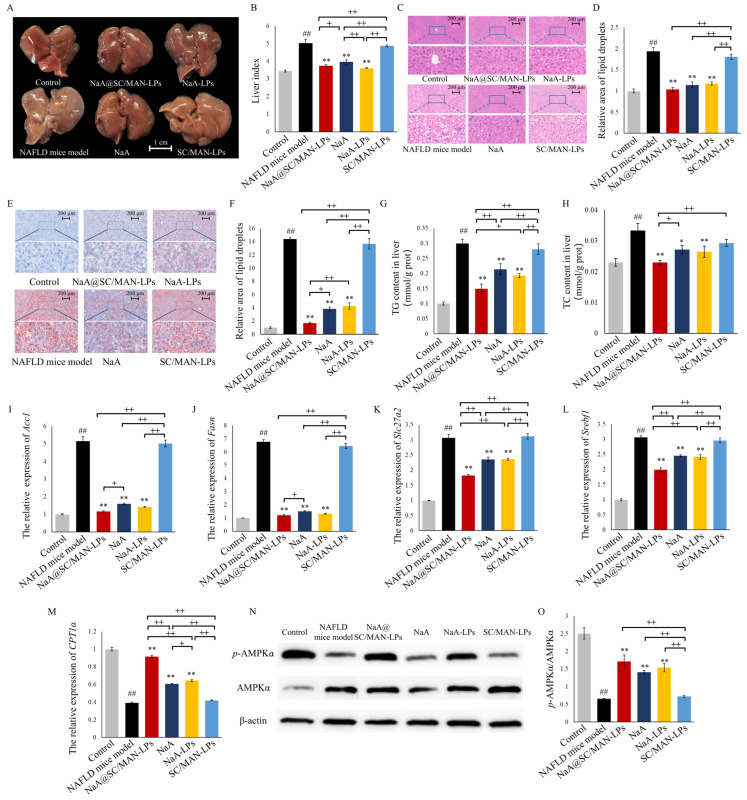
Effects of NaA liposomes or NaA on hepatic lipid accumulation and lipid accumulation-related mRNA expression in NAFLD mice. (**A**) The appearance of liver (scale bar = 1 cm); (**B**) the index of liver (%); (**C**) the H&E staining of liver tissue (100×, scale = 200 μm); (**D**) relative area of lipid droplets based on H&E staining of liver tissue (*n* = 8); (**E**) Oil red O staining of liver tissue (100×, scale bar = 200 μm); (**F**) relative area of lipid droplets based on Oil red O staining (*n* = 8); (**G**) hepatic triglyceride content; (**H**) hepatic cholesterol content; (**I**–**M**) the relative expression of *Acc1*, *Fasn*, *Slc27a2*, *Srebf1*, and *CPT1*α; (**N**) western blot image of AMPKα and *p*-AMPKα; (**O**) the relative expression of *p*-AMPKα/AMPKα. Data are mean ± SD. ^##^ *p* < 0.01, versus control group; * *p* < 0.05, ** *p* < 0.01, versus NAFLD group; ^+^ indicates a significance between treatment groups, ^+^ *p* < 0.05, ^++^ *p* < 0.01.

As shown in Figure 5A, the livers of the NaA@SC/MAN-LPs, NaA, and NaA-LPs groups regained a reddish-brown color and smooth surface, indicating NaA and NaA liposomes improved the liver morphology in NAFLD mice. In Figure 5B, the liver index of mice treated with NaA@SC/MAN-LPs, NaA, or NaA-LPs was significantly reduced compared to the NAFLD model group. Furthermore, NaA@SC/MAN-LPs and NaA-LPs significantly lower the liver index compared to NaA, indicating that NaA encapsulated in liposomes is more effective than NaA alone in improving liver pathology in NAFLD mice.

H&E and Oil Red O staining of liver sections was performed to assess the alleviation of hepatic pathology (Figure 5C–F). H&E results in NAFLD model mice showed disrupted liver lobular structure and ballooning degeneration of hepatocyte characterized by enlarged cells containing large lipid droplets, and some hepatocytes exhibited a mixed type of macrovesicular and microvesicular steatosis. After treatment with NaA@SC/MAN-LPs, NaA, or NaA-LPs, there was a significant decrease in the number and size of lipid vacuoles in the liver, indicating marked improvement in hepatic steatosis. Mice treated with SC/MAN-LPs exhibited a reduction in vacuole size but with a high number of vacuoles remaining, suggesting limited efficacy in reducing lipid deposition. Quantitative analysis showed that treatment with NaA@SC/MAN-LPs, NaA, or NaA-LPs significantly reduced lipid droplet area (*p* < 0.01), with reductions of 46.39%, 40.97%, and 39.33%, respectively. Oil Red O staining further showed that the livers of the NAFLD and SC/MAN-LPs groups had a significant increase in the size and number of lipid droplets, indicating substantial fat deposition in livers. Treatment with NaA@SC/MAN-LPs, NaA, and NaA-LPs significantly reduced lipid deposition, with NaA@SC/MAN-LPs demonstrating the most pronounced effect. These results suggest that NaA@SC/MAN-LPs show the most superior efficacy in reducing hepatic lipid droplet deposition compared to NaA-LPs and NaA alone. As shown in Figure 5G,H, after 10 weeks of treatment with NaA@SC/MAN-LPs, NaA, or NaA-LPs, both TG and TC levels in the liver were significantly reduced. NaA@SC/MAN-LPs treatment resulted in significantly lower liver TG and TC levels than NaA solution, further supporting the enhanced efficacy of dual-labeled liposomes in mitigating hepatic lipid deposition.

Gene expression analysis of lipid metabolism in the liver are shown in Figure 5I–M. The NAFLD model group exhibited significant upregulation of key genes involved in lipid biosynthesis (*Acc1*, *Fasn*, *Slc27a2*, and *Srebf1*) (*p* < 0.01) and a downregulation of *CPT1*α (*p* < 0.01), compared to the control group. Treatment with NaA@SC/MAN-LPs, NaA, or NaA-LPs significantly reduced the mRNA expression levels of *Acc1*, *Fasn*, *Slc27a2*, and *Srebf1*, while significantly upregulating *CPT1*α. These suggest that NaA or NaA-containing liposomes effectively alleviate the upregulation of hepatic lipid biosynthesis-related genes and promote the expression of the fatty acid transport in NAFLD mice. Furthermore, NaA@SC/MAN-LPs were more effective in decreasing *Acc1*, *Fasn*, *Slc27a2*, and *Srebf1* expression and increasing *CPT1*α expression compared to NaA alone, indicating that encapsulating NaA in SC and MAN dual-labeled liposomes enhanced the alleviation of hepatic lipid deposition in NAFLD mice. The primary pathogenesis of NAFLD is dysregulated lipid metabolism. AMPK is a key enzyme that regulates cellular metabolism and plays a crucial role in restoring lipid metabolic balance. As shown in Figure 5N,O, compared to the control group, AMPKα phosphorylation was significantly reduced in the livers of NAFLD mice and in the SC/MAN-LPs treatment group. However, after treatment with NaA@SC/MAN-LPs, NaA, or NaA-LPs, the phosphorylation level of AMPKα was significantly increased.

The observed changes in gene expression, consistent with protein expression results, showed that the administration of NaA@SC/MAN-LPs, NaA-LPs, or NaA significantly reduced the mRNA expression of *Acc1*, *Fasn*, *Slc27a2*, and *Srebf1c* while increasing the expression of *CPT1*α. These findings suggest that NaA@SC/MAN-LPs activate the AMPK pathway, inhibit the changes in gene expression of lipid metabolism, then lead to a reduction in hepatic lipid accumulation in NAFLD mice.

### 3.11. In Vivo Exhibition on Hepatic Oxidative and Inflammation in NAFLD Mice

**Figure 6:**
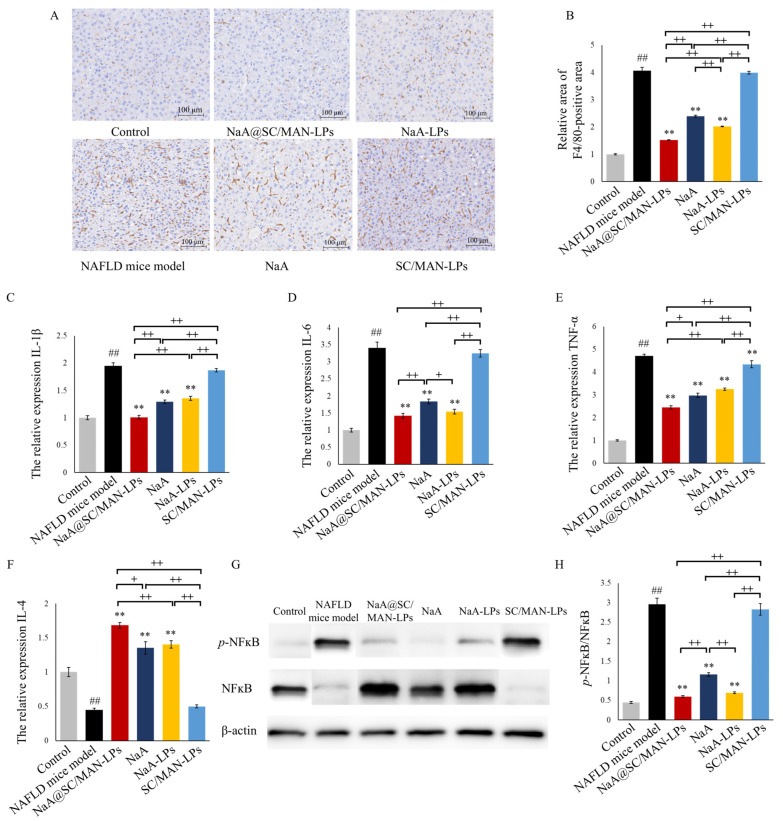
Effects of NaA liposomes or NaA on hepatic oxidative stress and inflammation in NAFLD mice. (**A**) F4/80 immunohistochemical staining of mouse liver (200×, scale bar = 100 μm); (**B**) relative area of F4/80-positive; (**C**–**F**) relative mRNA expression of inflammatory cytokines, IL-4, IL-6, IL-1β, TNF-α; (**G**) western blot image of *p*-NFĸB and NFĸB; (**H**) the relative expression of *p*-NFĸB/NFĸB. Data are mean ± SD. ^##^ *p* < 0.01, versus control group; ** *p* < 0.01, versus NAFLD group; ^+^ indicates a significance between treatment groups, ^+^ *p* < 0.05, ^++^ *p* < 0.01.

This study examines the effects of different NaA liposomes on alleviating liver inflammation in NAFLD mice, focusing on macrophage activation levels and mRNA expression of inflammation-related cytokines. F4/80 immunohistochemical staining was performed as shown in Figure 6A,B. Compared to the control group, the NAFLD model group exhibited a significant increase in the F4/80-positive staining area, indicating substantial macrophage infiltration in the liver. While the SC/MAN-LPs group did not show significant improvement in macrophage infiltration, while treatment with NaA@SC/MAN-LPs, NaA, or NaA-LPs led to considerable reductions in macrophage accumulation. Quantitative analysis of the immunohistochemical staining using ImageJ software revealed that after gavage with NaA@SC/MAN-LPs, NaA, or NaA-LPs, the relative F4/80-positive area in the liver decreased by 62.46%, 40.99%, and 50.16%, respectively. Among the treatment groups, NaA@SC/MAN-LPs was significantly more effective in alleviating macrophage infiltration than NaA solution or NaA-LPs (*p* < 0.01).

Further analysis was performed to assess the expression of inflammation and oxidative stress-related cytokines in the livers of NAFLD mice (Figure 6C–F). Compared to the control group, the NAFLD model group exhibited significantly higher mRNA levels of pro-inflammatory cytokines IL-1β, IL-6, and TNF-α, alongside a marked reduction in the anti-inflammatory cytokine IL-4, indicating a strong inflammatory response. However, treatment with NaA@SC/MAN-LPs, NaA, or NaA-LPs significantly reduced the mRNA expression of pro-inflammatory cytokines IL-1β, IL-6, and TNF-α, while significantly increasing IL-4 expression. Further comparison among the treatment groups revealed that NaA@SC/MAN-LPs were significantly more effective than NaA solution alone in reducing pro-inflammatory cytokine levels and increasing anti-inflammatory cytokine expression.

As shown in Figure 6G,H, in NAFLD mice, liver phosphorylation levels of NF-κB were significantly elevated compared to the control group. Treatment with SC/MAN-LPs alone did not significantly affect NF-κB phosphorylation. However, gavage with NaA@SC/MAN-LPs, NaA, or NaA-LPs significantly downregulated NF-κB phosphorylation, suggesting a reduction in HFD-induced liver inflammation. Furthermore, both labeled and unlabeled NaA liposomes were significantly more effective in reducing NF-κB phosphorylation than NaA solution alone.

## 4. Discussion

This study developed SC- and MAN-modified liposomes loaded with high concentrations of NaA, aimed at dual targeting hepatocytes and Kupffer cells. After oral administration, the NaA liposomes traverse the intestinal tract and enter the liver, significantly increasing the effective concentration and retention time of NaA in the liver, reducing lipid accumulation in hepatocytes, and inhibiting inflammation in Kupffer cells, while simultaneously improving both lipid accumulation and inflammation in NAFLD. This study presents the first design of a liposome formulation loaded with NaA that simultaneously targets both hepatocytes and Kupffer cells, offering a novel oral therapeutic approach for the treatment of NAFLD.

As an LMW compound, sodium acetate exhibits non-specific distribution in the body and poor pharmacokinetics, leading to off-target effects and low therapeutic efficacy. Studies have shown that sodium acetate is primarily absorbed in the distal colon [18]. Compared to proximal colon infusion, overweight males who received distal colon infusion of sodium acetate were able to more effectively increase fasting blood acetate levels. This was due to the high expression of SCFAs receptor, such as GPR43, in the distal colon and the lower conversion rate in the region, which thereby enhances acetate absorption. Intravenous administration of sodium acetate had confirmed a short in vivo metabolic half-life of acetate, which was only 9.3 ± 0.6 min [17]. Oral or gavage administration of acetate for NAFLD treatment has shown poor efficacy. One study reported that supplementing mice’s drinking water with 200 mM sodium acetate for 21 days resulted in no significant change in their blood acetate levels [36]. In another experiment, mice were gavaged with 1 g/Kg sodium acetate, and the result showed a short retention time of acetate in plasma, with the peak concentration reached at 15 min, which returned to baseline within 3 h [36]. Similar results were observed in human trials where healthy volunteers consumed 750 mg of vinegar, leading to peak serum acetate levels at 15 min, which returned to baseline after 90 min, with a maximum acetate concentration of 0.2 mM [37], far below the effective hepatic concentration required to alleviate NAFLD (2 mM) [14]. Furthermore, there are several issues associated with oral sodium acetate supplementation. It has an unpleasant odor, poor palatability, poor stability in the gastrointestinal environment, low bioavailability after systemic distribution, and it is prone to degradation in the acidic conditions of the stomach or hydrolysis by mucosal proteins [19]. High concentrations of SCFAs may also cause gastrointestinal damage, including erythema, bleeding, ulcers, and peritonitis [20]. Therefore, there is an urgent need to develop an oral delivery system for NaA to improve its bioavailability and therapeutic efficacy.

Currently, drug delivery systems targeting the liver are primarily used for hydrophobic drugs (such as silymarin and curcumin) or macromolecular drugs (such as doxorubicin and berberine hydrochloride) [28,38]. For SCFA delivery, nano-delivery systems including polymer nanoparticles, SCFA self-assembled micelles, and liposomes were reported in previous studies. For the polymer nanoparticles, the carboxyl group of butyrate interacts electrostatically with the positive amino group of chitosan, exhibiting a sustained release of butyrate and continuously inhibiting reactive oxygen species (ROS) release by neutrophils [39]. However, maintaining structural stability in vivo remains challenging. SCFA self-assembled micelles are formed by combining polyethylene glycol and vinyl SCFA ester into an amphiphilic block copolymer, which self-assembles into micelles [40]. After oral administration, these micelles are hydrolyzed by digestive enzymes and enter the bloodstream. However, the material, vinyl SCFA ester, is toxic and lacks organ targeting after entering circulation. Liposomes, with their hydrophilic core and easily modifiable surface, offer advantages in the targeted delivery of water-soluble small molecules such as acetate [32]. Typically, specific ligands are modified on the surface of nanoparticles to achieve targeted drug delivery. Sodium cholate and mannose are common liver-targeting ligands. Sodium cholate specifically binds to the NTCP receptor’s high expression on the surface of hepatocytes, while mannose specifically targets the mannose receptor’s high expression on Kupffer cells. Oral nanoparticle liposomes with particle sizes between 100 and 150 nm prevent the activation of the mononuclear phagocytic system and extend circulation time [41,42]. Surface modification with polyethylene glycol (PEG) improves mucus permeability for oral drug delivery [43]. Cationic liposomes have greater stability during gastrointestinal fluid and storage conditions [44,45]. Since the intestinal mucus layer is negatively charged, cationic liposomes are more readily absorbed into the phospholipid bilayer, increasing their retention time in the intestine and prolonging drug absorption time [46]. Currently, SCFA-loaded liposomes release SCFAs in the intestine, which are absorbed via the portal vein and distributed systemically without liver-targeted delivery. As shown by in vitro and in vivo experiments, SC- and MAN-modified NaA liposomes have no significant toxicity. The intervention period of this study demonstrated that NaA liposomes exhibited no physiological toxicity. After 10 weeks of NaA liposome supplementation, liver lipid deposition and inflammatory infiltration in NAFLD mice did not worsen, further confirming the safety of the liposomes. Additionally, the materials used in liposomes, phospholipids and sterols, are well established as safe, and liposomal drug formulations have been approved for clinical treatment [47,48,49]. Therefore, NaA liposomes are considered biocompatible. The biological safety of longer-term supplementation of NaA liposomes needs to be verified in different animal models and human trials.

The pathological features of NAFLD are primarily characterized by lipid accumulation in hepatocytes and inflammatory infiltration of macrophages [50]. Lipid accumulation in hepatocytes induces lipotoxicity, leading to hepatic steatosis, which triggers the release of stress signals and activates inflammatory pathways, resulting in liver inflammation. Recent studies have shown that the immune activation of Kupffer cells is a key factor in initiating and exacerbating liver inflammation in NAFLD. Upon activation, Kupffer cells secrete pro-inflammatory cytokines (such as TNF-α, IL-1β, IL-6), promoting the progression of NAFLD. Moreover, they recruit neutrophils and other inflammatory cells, enhancing insulin resistance and inflammation and potentially triggering fibrosis [51,52]. The results of the NAFLD population trial indicated a significant reduction in acetate content compared to healthy individuals [7]. However, data from another clinical study showed an increase in acetate content in the feces of patients with advanced liver fibrosis [53]. This discrepancy may be attributed to the varying severity of NAFLD among patients, as well as differences in factors such as region, age, and the method used to detect SCFAs. Nevertheless, numerous studies have confirmed that NaA supplementation is effective in alleviating lipid deposition and inflammation in NAFLD [5]. Acetate effectively alleviates NAFLD by reducing lipid accumulation in hepatocytes and attenuating inflammatory responses in Kupffer cells. It promotes AMPKα phosphorylation, upregulates the expression of lipid oxidation genes, and reduces the expression and transcriptional activity of SREBP-1c, inhibiting lipogenesis [11]. For inhibiting inflammation, it modulates Kupffer inflammatory activation through the PPARγ/UCP2/AMPK/iNOS/IκBα/NF-κB signaling pathway [14]. Studies have shown that high doses of sodium acetate reduce hepatic lipid accumulation by inhibiting Kupffer cell inflammation [14]. The target cells for alleviating NAFLD are hepatocytes and Kupffer cells. And the effective concentration of sodium acetate in the liver is a critical factor for the therapeutic effects of sodium acetate in alleviating NAFLD.

In this study, we constructed a cationic liposome with an approximate diameter of 100 nm, incorporating PEG components with mucus penetration and hydrophilic properties into the basic lipid framework. By optimizing the ratio of DSPE and cholesterol, we achieved efficient encapsulation and transport of high concentrations of NaA. Furthermore, we developed a liver-targeted delivery system by modifying NaA acetate liposomes with DSPE-PEF-SC and DSPE-PEF-MAN, enabling active targeting of NaA to the liver via SC/NTCP and MAN/MAN receptors. In vivo distribution experiments of the liposomes demonstrated that the acetate concentration of SC-MAN-dual modified liposomes accumulated in the liver was 3.75 times more than NaA alone. Compared to oral administration of NaA alone, oral administration of NaA@SC/MAN-LPs increased the maximum concentration of acetate in the liver. Additionally, the half-life of acetate in the liver increased from 2.85 h to 15.58 h. These NaA-loaded and dual-modified liposomes significantly enhancing the hepatic retention time and effective concentration of NaA. The endocytosis pathway of NaA@SC/MAN-LPs by cells are marcopinocytosis, clathrin-mediated endocytosis, SC-ASBT/NTCP receptor-mediated endocytosis, and MAN/MAN receptor-mediated endocytosis. To the best of our knowledge, these liposomes represent the first successful achievement of liver-targeted delivery of NaA after oral administration.

The NaA@SC/MAN-LPs demonstrated excellent efficacy in alleviating NAFLD in mice. NaA@SC/MAN-LPs significantly reduced body weight gain, improved glucose tolerance, and lowered hepatic levels of TG, TC, and LDL-c in HFD-induced NAFLD mice. Apart from differences in metabolic rates, there are no substantial disparities in the metabolism and function of signaling molecules between mice and humans [54]. In this study, the dual-modified NaA liposomes used in the animal experiments of this study showed promising results. Therefore, NaA liposomes have potential for clinical application and represent a novel strategy for the treatment of NAFLD.

## 5. Conclusions

This study synthesized NaA-loaded liposomes modified by SC and MAN (NaA@SC/MAN-LPs), which demonstrated both intestinal penetration and liver-targeting capabilities after oral administration. The NaA liposome effectively increased the concentration of acetate in the liver and prolonged its hepatic retention time. The liposomes selectively targeted two key cells involved in NAFLD, hepatocytes and Kupffer cells. It not only activated AMPK signaling phosphorylation, which reduced lipid accumulation in hepatocytes, but also inhibited NFκB phosphorylation, suppressed Kupffer cell inflammatory responses, and alleviated liver inflammation. NaA@SC/MAN-LPs represent an efficient and safe NaA delivery system, demonstrating significant potential for alleviating NAFLD and offering a novel approach for the oral treatment of NAFLD. Further research should focus on elucidating the intracellular release mechanisms after targeted delivery and validating its efficacy in clinical trials to confirm its ability to mitigate NAFLD in human populations.

## Figures and Tables

**Table 1:** The characterization of five NaA liposomes.

Group	Partial Size (nm)	PDI	Zeta Potential (mV)	EE%	DL%
NaA@SC/MAN-LPs	94.15 ± 0.23	0.23 ± 0.01	+33.00 ± 3.71	76.6 ± 0.47	33.16 ± 0.16
NaA@SC-LPs	100.46 ± 1.56	0.24 ± 0.01	+33.10 ± 3.56	81.57 ± 0.94	37.94 ± 0.36
NaA@MAN-LPs	98.35 ± 1.55	0.24 ± 0.02	+32.40 ± 6.19	78.90 ± 0.07	33.29 ± 0.03
NaA@LPs	95.29 ± 1.07	0.22 ± 0.01	+27.50 ± 5.06	79.03 ± 0.30	35.28 ± 0.26
SC/MAN-LPs	96.53 ± 0.68	0.23 ± 0.00	+33.00 ± 3.71	-	-

## Data Availability

The original contributions presented in the study are included in the article/Supplementary Material, further inquiries can be directed to the corresponding author.

## Supplementary Materials

The following supporting information can be downloaded at https://www.mdpi.com/article/10.3390/nu17050930/s1. Figure S1: DSC thermogram and FTIR spectra of DSPE-PEG^2000^-SC and DSPE-PEG2^000^-MAN; Figure S2: The release of NaA from liposomes after incubation in serum (A), SGF (B), and SIF (C); Figure S3: EE% of NaA (A) and DL% of nine liposomes (B) at different NaA stock solution concentrations; Figure S4: Effects of acetate liposomes on fat content of adipose tissue; Table S1: The optimization factors and levels of NaA liposomes; Table S2: The orthogonal experiment group table; Table S3: Weight ratios and energy rations for the maintenance feed and the HFD; Table S4: Sequences of primer used for RT-qPCR; Table S5: Effects of liposome compositions on the EE% and DL%; Table S6: Particle size, PDI and zeta potential variations of liposomes in serum, SGF, and SIF; Table S7: ^13^C-acetic acid pharmacokinetic characteristics in ^13^C-NaA@SC/MAN-LPs, ^13^C-NaA-LPs, and ^13^C-NaA group mice; Table S8: ^13^C-acetic acid metabolism characteristics in serum, liver, cecum, and colon; Table S9: Storage stability of five types of liposomes at 4 °C.

## Abbreviations

The following abbreviations are used in this manuscript:

NaA	sodium acetate
NAFLD	non-alcoholic fatty liver disease
SC	sodium cholate
MAN	mannose
TG	triglycerides
TC	total cholesterol
NASH	non-alcoholic steatohepatitis
HCC	hepatocellular carcinoma
SCFAs	short-chain fatty acids
AMPKα	AMP-activated protein kinase alpha
FFAR2	free fatty acid receptor 2
LPS	lipopolysaccharide
HFD	high-fat diet
GLP-1	glucagon-like peptide 1
PYY	peptide YY
LMW	low-molecular-weight
NTCP	Na^+^-taurocholate co-transporting polypeptide
ASBT	bile acid-na^+^-dependent bile acid transporter
DSPE-PEG^2000^-NH_2_	1,2-distearoyl-sn-glycero-3-phosphoethanolamine-*N*-[amino(polyethylene gly-col)-2000]
DSPC	1,2-dioctadecanoyl-sn-glycero-3-phosphocholine
DC-cholesterol	3β-[*N*-(Dimethylaminoethane) carbamoyl] cholesterol
EDC	*N*-Ethyl-*N*′-(3-dimethylaminopropyl)carbodiimide
NHS	*N*-Hydroxysuccinimide
CY7-SE triethylamine	sulfo-cyanine7 succinimidyl ester triethylamine
DMSO	dimethyl sulfoxide
HPLC	high-performance liquid chromatography
AM	amiloride hydrochloride
CH	chlorpromazine
M-β-CD	Me-Thyl-β-Cyclodextrin
GE	genistrin
NY	nystatin
GC-MS	triple quadrupole gas chromatography–mass spectrometry
DSC	differential scanning calorimeter
FTIR	Fourier transform infrared spectrometer
PDI	poly-dispersity indexes
EE	encapsulation efficiency
DL	drug loading
TEM	transmission electron microscopy
OL	sodium oleate
PA	sodium palmitate
Acc1	acetyl-CoA carboxylase 1
Fasn	atty acid synthase
Scd1	stearoyl-CoA desaturase 1
Srebf1	sterol regulatory element-binding transcription factor 1
CPT1α	carnitine palmitoyltransferase 1α
OGTT	oral glucose tolerance tests
LDL-C	low-density lipoprotein cholesterol
HDL-C	high-density lipoprotein cholesterol
ALT	alanine aminotransferase
AST	aspartate aminotransferase
ROS	reactive oxygen species
