# Supplementary material for: Targeted Sodium Acetate Liposomes for Hepatocytes and Kupffer Cells: An Oral Dual-Targeted Therapeutic Approach for Non-Alcoholic Fatty Liver Disease Alleviation
Source: Nutrients. 2025 Mar 6;17(5):930. doi: 10.3390/nu17050930 (PMC11901740; doi:10.3390/nu17050930)
Supplement: Supplementary file 1 [file nutrients-17-00930-s001.zip › nutrients-3489249-supplementary.pdf]

Supplementary Materials

Targeted Sodium Acetate Liposomes for Hepatocytes and Kupffer Cells: An Oral Dual-Targeted Therapeutic Approach for Non-Alcoholic Fatty Liver Disease Alleviation

Yichao Hou [1], Xilong Gao [1], Jiahui Gong [2], Xinrui Dong [2], Yanling Hao [1], Zhengyuan Zhai [2], Hao Zhang [2], Ming Zhang [3], Rong Liu [1], Ran Wang [1,4] and Liang Zhao [1,2,4,*]

[1] Key Laboratory of Functional Dairy, Department of Nutrition and Health, China Agricultural University, Beijing 100193, China
[2] College of Food Science and Nutritional Engineering, China Agricultural University, Beijing 100083, China
[3] School of Food and Health, Beijing Technology and Business University, Beijing 100048, China
[4] Research Center for Probiotics, China Agricultural University, Beijing 101299, China
[*] Correspondence: lzhao@cau.edu.cn

Results

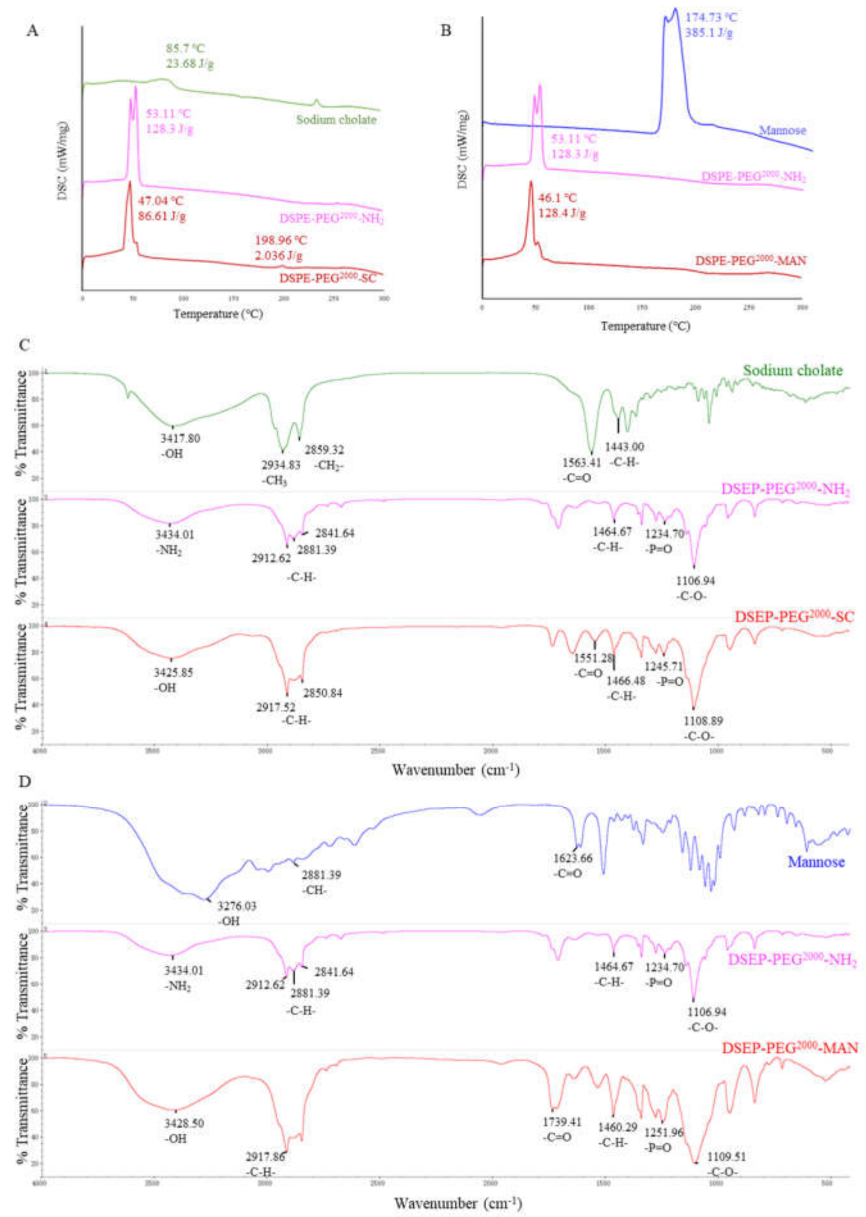

Figure S1. Differential scanning calorimetry (DSC) thermogram and Fourier transform infrared spectroscopy (FTIR) spectra of DSPE-PEG^{2000}-SC and DSPE-PEG^{2000}-MAN.

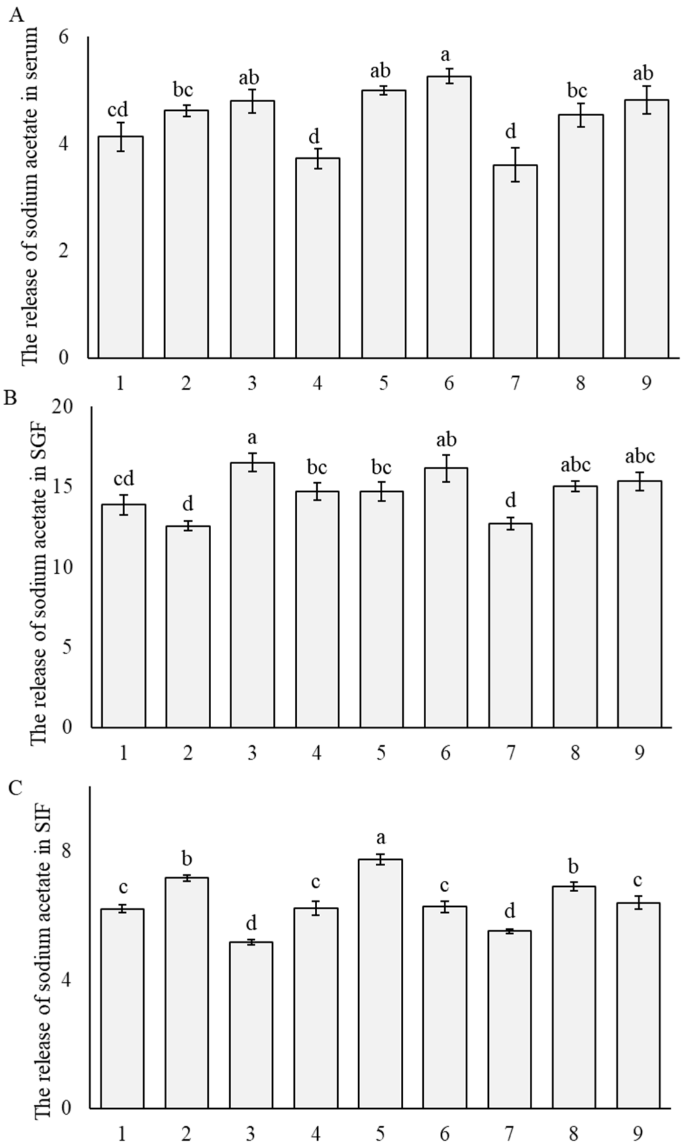

Figure S2. The release of sodium acetate from liposomes after incubation in serum (**A**), SGF (**B**) and SIF (**C**). Data represent mean ± SD. Different letters indicate significant differences ($p<0.05$), while the same letter indicates no significant difference ($p>0.05$).

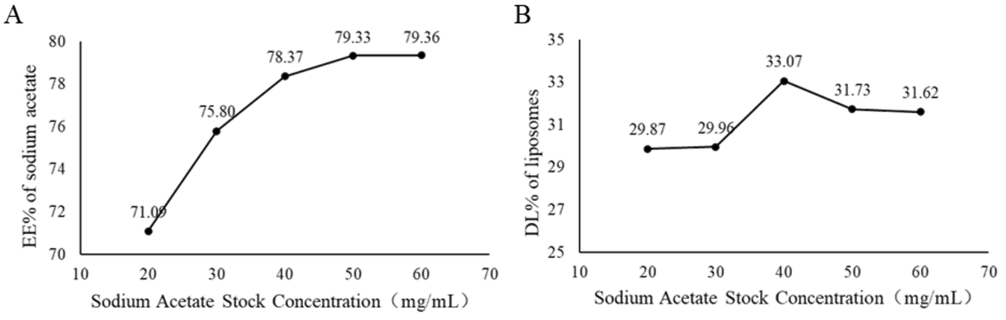

Figure S3. EE% of sodium acetate (**A**) and DL% of nine liposomes (**B**) at different sodium acetate stock solution concentration.

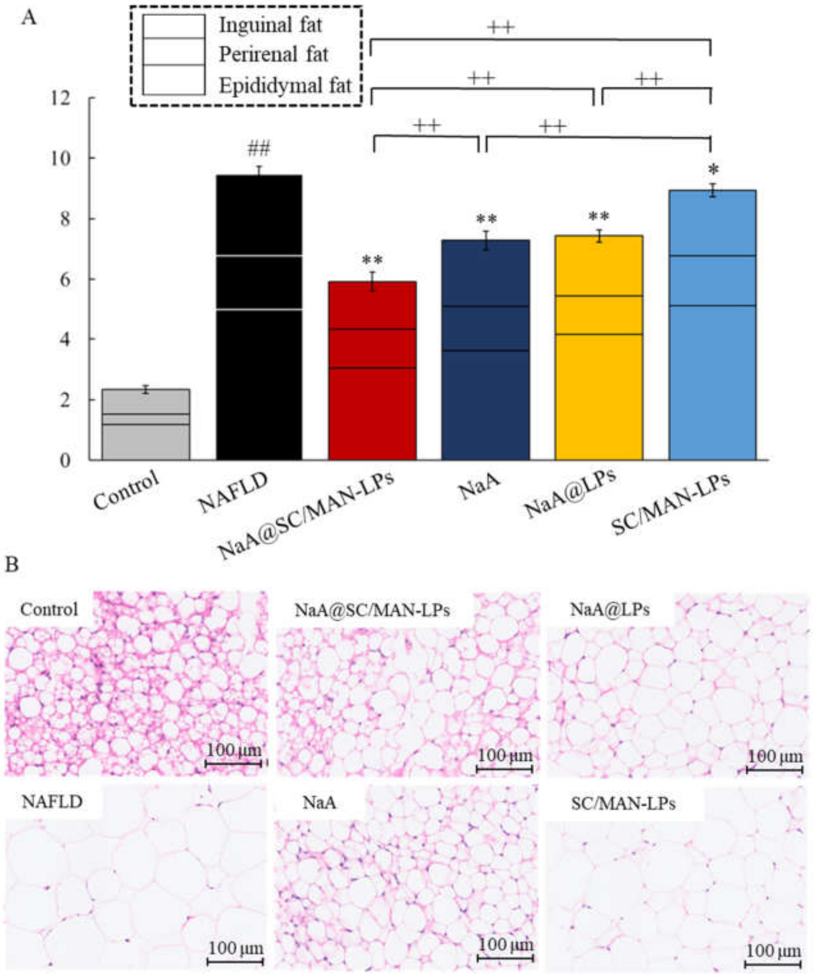

Figure S4 Effects of acetate liposomes on fat content of adipose tissue. (**A**) The proportion of white adipose tissue to body weight; (**B**) H&E staining of epididymal visceral adipose tissue (400×, scale bar=100 μm); $^{##}p$<0.01, versus control group; $^{*}p$<0.05, $^{**}p$<0.01, versus NAFLD group; $^{+}$indicates a significance between treatment groups, ^{++}p<0.01.

METHODS

Synthesis of DSPE-PEG^{2000}-SC and DSPE-PEG^{2000}-MAN

Cholic acid, EDC, and NHS were dissolved in dichloromethane and reacted in an ice bath for 30 minutes, followed by stirring at room temperature overnight. The mixture was filtered and rotary evaporated to obtain activated sodium cholate. DSPE-PEG^{2000}-NH_2 (500 mg) was weighed and dissolved in 3 mL of DMSO. Actived sodium cholate (1.1 eq.) and triethylamine (2.0 eq.) were added to completely dissolve the solution. The solution was reacted for 2 h at 40°C. The reaction solution was transferred to a dialysis bag with a molecular weight cutoff of 1000 Da. The solution was dialyzed in pure water for 24 h. The dialysate was collected and freeze-dried to obtain DSPE-PEG^{2000}-SC.

DSPE-PEG^{2000}-NH_2 (500 mg) was weighed and dissolved in 3 mL of DMSO. Mannose (1.1 eq.) and triethylamine (2.0 eq.) were added to completely dissolve the solution. The solution was reacted for 2 h at 40 ^{o}C. The reaction solution was transferred to a dialysis bag with a molecular weight cutoff of 1,000 Da. The solution was dialyzed in pure water for 24 h. The dialysate was collected and freeze-dried to obtain DSPE-PEG^{2000}-MAN.

Preparation of NaA-loaded liposomes

Liver-targeted sodium acetate liposomes for oral delivery were prepared using the thin-film hydration method. All reagents were stored at -20 ^{o}C, and the preparation process was conducted on ice. The liposome formulation including the following components: DSPC, cholesterol, DSPE-PEG^{2000}-SC, DSPE-PEG^{2000}-MAN, DSPE-PEG^{2000} and DC-cholesterol. The mixed solution was shaken in a constant-temperature shaker at 37 ^{o}C for 10 minutes to ensure homogeneous thermodynamic distribution of the reagents. Rotary evaporation was performed under a pressure of 500 mbar and a temperature of 50 ^{o}C. Subsequently, a sodium acetate stock solution was added to fully immerse the organic membrane. The mixture was then subjected to ultrasonic for 2 minutes, resulting in a milky-white solution. Liposomes were prepared using an extrusion apparatus (Avanti Polar Lipids) with 100 nm filter membrane. Unencapsulated sodium acetate was removed via ultrafiltration, after which the liposomes were rapidly frozen in liquid nitrogen.

The optimization experiment of sodium acetate loaded liposomes

The sodium acetate liposomes were consisted of DSPC, cholesterol, DSPE-PEG^{2000}-SC, DSPE-PEG^{2000}-MAN, DSPE-PEG^{2000}, and DC-cholesterol. To achieve high encapsulation efficiency (EE%) of sodium acetate and stability, the ratios of DSPC to cholesterol, DSPE-PEG^{2000}-SC, and DSPE-PEG^{2000}-MAN were optimized using an orthogonal experimental design. The concentration of the sodium acetate stock solution was fixed at 20 mg/mL. The optimization factors and levels for the composition of sodium acetate liposomes are presented in Table S1. The orthogonal experiment groups are also shown in Table S2.

Table S1. The optimization factors and levels of sodium acetate liposomes.

Factors	Levels		
DSPC : cholesterol (mol/mol)	1:1	2:1	3:1
DSPE-PEG-SC (M/M %)	1%	5%	10%
DSPE-PEG-MAN (M/M %)	1%	5%	10%

Table S2. The orthogonal experiment group table.

Group	DSPC	cholesterol	DSPE-PEG2000-SC	DSPE-PEG2000-MAN	DSPE-PEG2000	DC-cholesterol
1	31.5%	31.5%	1 %	1%	5%	30%
2	27.5%	27.5%	5%	5%	5%	30%
3	22.5%	22.5%	10%	10%	5%	30%
4	39.3%	19.7%	1%	5%	5%	30%
5	33.3%	16.7%	5%	10%	5%	30%
6	36%	18%	10%	1%	5%	30%
7	40.5%	13.5%	1%	10%	5%	30%
8	44.25%	14.75%	5%	1%	5%	30%
9	37.5%	12.5%	10%	5%	5%	30%

Following the optimization of liposome composition ratios, the concentration of sodium acetate stock solution was further optimized to maximize the EE% and DL% of sodium acetate in the liposomes. Sodium acetate stock solutions were prepared at concentrations of 20, 30, 40, 50, and 60 mg/mL. Subsequently, NaA@SC/MAN-LPs were prepared according to the optimized liposome formulation. The concentration of sodium acetate was measured using HPLC to calculate the EE%. The liposomes were then rapidly frozen with liquid nitrogen, lyophilized, and weighed to determine the DL% of sodium acetate based on liposome mass. The optimal concentration of sodium acetate stock solution was determined based on the DL% and EE% across the different stock concentrations.

Determination of sodium acetate concentration by HPLC

The HPLC conditions were as follows: a Thermo Hypersil ODS-2 C18 column (4.6 mm × 200 mm, 5 μm) was utilized. The mobile phase consisted of 0.01 mol/L ammonium dihydrogen phosphate aqueous solution, with the pH adjusted to 3.0 using 1 mol/L phosphoric acid. The flow rate was set at 1.0 mL/min, with an injection volume of 10 μL. Detection was carried out at a wavelength of 215 nm, and the column temperature was maintained at 30 ^{o}C.

The sodium acetate liposome solution was placed in an activated ultrafiltration tube (MWCO = 3K Da, Millipore) and centrifuged at 12,000 rpm for 10 minutes. The filtrate was collected, and the free sodium acetate content was determined by HPLC. The EE% of sodium acetate in the liposomes was then calculated using the following formula: EE%=(Total mass of sodium acetate- Mass of free sodium acetate)/Total mass of sodium acetate ×100%. The calculation formula for drug loading (DL%) was as follows: DL% = Mass of encapsulated sodium acetate in liposomes/ Total mass of liposomes×100%.

Stability in simulated body fluid

The stability of sodium acetate liposomes in different simulated body fluids was evaluated. Sterile fetal bovine serum was diluted tenfold with PBS (pH 7.4) to prepare the simulated serum solution. Simulated gastric fluid (SGF) was prepared by dissolving 0.5 g of pepsin in 10 mL of pure water, followed by the addition of 0.82 mL of 1 mol/L hydrochloric acid solution. The mixture was thoroughly mixed and diluted to 50 mL, with the pH adjusted to 1.2. Simulated intestinal fluid (SIF) was prepared by dissolving 0.34 g of potassium hydrogen phosphate in 25 mL of water, adjusting the pH to 6.8 using 0.4% NaOH, and then dissolving 0.5 g of trypsin in water. The two solutions were mixed and diluted to 50 mL.

A 100 μL aliquot of sodium acetate liposomes was mixed with 900 μL of SGF, SIF, or serum solution, and incubated at 37 ^{o}C with shaking at 1000 rpm. Samples were collected from SGF solution after 2 hours, and from SIG or serum solution after 6 hours. The particle size, PDI, and Zeta potential of sodium acetate liposomes were measured using a laser particle size analyzer, while the sodium acetate release rate in different media was determined by HPLC. The stability of the liposomes in serum, SFG and SIG was evaluated by analyzing changes in phenotypes, EE% and DL% before and after incubation.

Storge stability of liposomes

The storage stability of five different modified sodium acetate liposomes (NaA@SC/MAN-LPs, NaA@SC-LPs, NaA@MAN-LPs, NaA@LPs, SC/MAN-LPs) was evaluated. The five liposome solutions with a concentration of 50 mg/mL were stored at 4 ^{o}C for 3 weeks, and the particle size, PDI and zeta potential of each group of liposomes were measured at 0, 1, 2 and 3 weeks using a Malvern particle sizer to evaluate the storage stability of liposomes.

Mouse feed energy in animal experiments

Table S3. Weight ratios and energy rations for the maintenance feed and the HFD.

Ingredient	Normal diet		HFD	
	g%	kcal%	g%	kcal%
Protein	19.2	20	26.2	20
Carbohydrate	67.3	70	26.3	20
Fat	4.3	10	34.9	60
Total		100		100
Kcal/g	3.85		5.24	

qRT-PCR

The primers related to lipid metabolism and inflammatory responses were sourced from NCBI (https://www.ncbi.nlm.nih.gov/) and PrimerBank (https://pga.mgh.harvard.edu/primerbank/). Genes were synthesized by Shanghai Shenggong Biological Engineering Company.

Table S4 Sequences of primer used for RT-Qpcr.

Genes	Forward（5′ to 3′）	Reverse（3′ to 5′）
Acc1	GATGAACCATCTCCGTTGGC	GACCCAATTATGAATCGGGAGTG
Fasn	AGGTGGTGATAGCCGGTATGT	TGGGTAATCCATAGAGCCCAG
Scd1	TTCTTGCGATACACTCTGGTGC	CGGGATTGAATGTTCTTGTCGT
Srebf1	GCAGCCACCATCTAGCCTG	CAGCAGTGAGTCTGCCTTGAT
Cpt1α	TGGCATCATCACTGGTGTGTT	GTCTAGGGTCCGATTGATCTTTG
IL-1β	GCAACTGTTCCTGAACTCAACT	ATCTTTTGGGGTCCGTCAACT
IL-4	GGTCTCAACCCCCAGCTAGT	GCCGATGATCTCTCTCAAGTGAT
IL-6	TAGTCCTTCCTACCCCAATTTCC	TTGGTCCTTAGCCACTCCTTC
TNF-α	GACGTGGAACTGGCAGAAGAG	TTGGTGGTTTGTGAGTGTGAG
Slc27a2	TCCTCCAAGATGTGCGGTACT	TAGGTGAGCGTCTCGTCTCG
Gapdh	GTCTTCACCACCATGGAGAA	TAAGCAGTTGGTGGTGCAG

Results

Table S5. Effects of liposome compositions on the EE% and DL%.

Group	EE% of sodium acetate	DL% of liposomes
1	77.71±0.47 [b]	27.46±0.16 [c]
2	73.41±0.94 [e]	27.81±0.36 [c]
3	81.15±0.07 [a]	31.58±0.03 [a]
4	76.09±0.80 [d]	25.62±0.27 [d]
5	70.52±0.87 [f]	20.86±0.26 [g]
6	71.28±0.58 [f]	23.22±0.19 [f]
7	78.42±0.30 [b]	29.70±0.11 [b]
8	76.89±0.64 [cd]	24.64±0.21 [e]
9	73.00±0.36 [e]	24.58±0.12 [e]

Data are mean ± SD. Different letters indicate significant differences ($p<0.05$), while the same letter indicates no significant difference ($p>0.05$).

Table S6. Particle size, PDI and Zeta potential variations of liposomes in serum, SGF, and SIF.

group		1	2	3	4	5	6	7	8	9
Particle Size	Initial	100.83±0.64 ^{a}	102.17±1.20 ^{a}	101.44±1.85 ^{a}	101.73±0.47 ^{a}	100.56±1.20 ^{a}	100.77±0.12 ^{a}	100.05±0.50 ^{a}	101.47±0.40 ^{a}	101.47±0.06 ^{a}
	Serum	395.85±8.44 ^{d}	173.07±2.03 ^{e}	527.41±6.23 ^{b}	428.14±11.77 ^{c}	393.42±6.54 ^{d}	177.80±3.36 ^{e}	132.41±3.67 ^{f}	177.60±1.46 ^{e}	644.77±1.82 ^{a}
	SGF	185.34±3.12 ^{c}	186.33±6.73 ^{c}	157.40±2.29 ^{d}	228.64±7.87 ^{b}	282.11±8.17 ^{a}	194.1±1.82 ^{c}	107.21±2.40 ^{e}	176.40±2.87 ^{cd}	128.70±1.59 ^{e}
	SIF	272.90±6.87 ^{cd}	319.33±7.08 ^{b}	600.27±5.53 ^{a}	253.76±8.18 ^{d}	282.55±3.13 ^{cd}	280.24±2.88 ^{cd}	279.77±7.22 ^{cd}	265.83±3.25 ^{cd}	283.37±5.53 ^{c}
PDI	Initial	0.21±0.02 ^{a}	0.21±0.02 ^{a}	0.21±0.01 ^{a}	0.20±0.01 ^{a}	0.20±0.00 ^{a}	0.20±0.00 ^{a}	0.20±0.02 ^{a}	0.21±0.01 ^{a}	0.21±0.01 ^{a}
	Serum	0.27±0.03 ^{a}	0.28±0.02 ^{a}	0.28±0.02 ^{a}	0.28±0.01 ^{a}	0.26±0.02 ^{a}	0.26±0.02 ^{b}	0.22±0.01 ^{b}	0.28±0.02 ^{b}	0.24±0.02 ^{b}
	SGF	0.29±0.01 ^{a}	0.26±0.01 ^{abc}	0.29±0.00 ^{ab}	0.24±0.01 ^{abc}	0.25±0.01 ^{abc}	0.27±0.03 ^{ab}	0.20±0.01 ^{c}	0.23±0.02 ^{abc}	0.22±0.00 ^{bc}
	SIF	0.28±0.00 ^{a}	0.26±0.01 ^{ab}	0.22±0.01 ^{b}	0.26±0.01 ^{ab}	0.26±0.01 ^{ab}	0.25±0.01 ^{ab}	0.23±0.01 ^{b}	0.25±0.01 ^{ab}	0.28±0.01 ^{a}
Zeta potential	Initial	30.70±6.27 ^{a}	29.30±6.32 ^{a}	18.80±6.69 ^{b}	29.40±4.71 ^{a}	20.20±4.05 ^{a}	29.40±3.36 ^{a}	37.60±4.18 ^{a}	36.30±3.45 ^{a}	16.90±3.02 ^{b}
	serum	10.10±3.17 ^{b}	10.60±3.53 ^{ab}	17.20±3.81 ^{a}	12.30±2.84 ^{ab}	10.10±3.34 ^{b}	7.62±3.41 ^{b}	7.26±2.68 ^{b}	7.96±6.01 ^{b}	9.10±3.2 ^{b}
	SGF	23.80±3.52 ^{a}	10.20±3.21 ^{b}	12.30±2.84 ^{b}	12.90±3.97 ^{b}	11.20±2.85 ^{b}	9.29±3.84 ^{b}	7.72±3.05 ^{b}	8.56±3.68 ^{b}	8.05±3.93 ^{b}
	SIF	22.3±3.3 ^{ab}	29.3±6.32 ^{a}	18.8±6.69 ^{ab}	15.3±3.72 ^{b}	16.3±4.11 ^{b}	16.3±3.78 ^{b}	12.2±3.42 ^{b}	11.7±3.49 ^{b}	11.9±2.9 ^{b}

Data are mean ± SD. Different letters indicate significant differences ($p<0.05$), while the same letter indicates no significant difference ($p>0.05$).

Table S7. ^{13}C-acetic acid pharmacokinetic characteristics in ^{13}C-NaA@SC/MAN-LPs, ^{13}C-NaA-LPs and ^{13}C-NaA group mice.

Pharmacokinetic characteristics	^{13}C-NaA@SC/MAN-LPs	^{13}C-NaA@LPs	^{13}C-NaA
C_{max} (μg/mL)	180.31±1.64 [a]	141.32±2.44 [b]	146.07±1.22 [b]
T_{max} (min)	30	30	15
AUG_{0-18} (μg·h/mL)	82843.19 [a]	32477.28 [b]	29252.29 [b]
$AUG_{0-\infty}$ (μg·h/mL)	86895.11 [a]	33169.36 [b]	29853.13 [b]
K_e	0.04	0.20	0.24
$T_{1/2}$ (h)	15.58	3.39	2.85

Data are mean ± SD. Different letters indicate significant differences (p<0.05), while the same letter indicates no significant difference (p>0.05).

Table S8. ^{13}C-acetic acid metabolism characteristics in serum, liver, cecum and colon.

		^{13}C-NaA@SC/MAN-LPs	^{13}C-NaA-LPs	^{13}C-NaA
Serum	C_{max} (μg/mL)	180.31±1.64 [a]	141.32±2.44 [b]	146.07±1.22 [b]
	T_{max} (min)	30	30	15
	AUG_{0-18} (μg·h/mL)	82843.19±194.34 [a]	32477.28±172.63 [b]	29252.29±132.82 [b]
Liver	C_{max} (μg/mL)	2.06±0.02 [a]	1.47±0.06 [b]	1.48±0.07 [b]
	T_{max} (min)	60	30	15
	AUG_{0-18} (μg·h/mL)	505.08±2.68 [a]	161.60±2.18 [b]	134.71±6.59 [b]
Cecum	C_{max} (μg/mL)	1.13±0.01 [c]	1.46±0.02 [b]	1.68±0.05 [a]
	T_{max} (min)	600	360	60
	AUG_{0-18} (μg·h/mL)	599.13±5.33 [b]	1005.88±2.31 [a]	1093.3±1.79 [a]
Colon	C_{max} (μg/mL)	1.34±0.02 [c]	1.48±0.08 [b]	2.63±0.04 [a]
	T_{max} (min)	600	360	30
	AUG_{0-18} (μg·h/mL)	546.24±1.49 [c]	854.33±6.13 [b]	950.75±3.38 [a]

Data are mean ± SD. Different letters indicate significant differences (p<0.05), while the same letter indicates no significant difference (p>0.05).

Table S9. Storage stability of five types of liposomes at 4 °C.

		0 week	1 week	2 weeks	3 weeks
Size (nm)	NaA@SC/MAN-LPs	101.13 ± 1.71 [a]	103.90 ± 2.11 [a]	104.52 ± 1.45 [a]	107.80 ± 2.51 [a]
	NaA@SC-LPs	104.18 ± 0.60 [a]	105.26 ± 0.37 [a]	105.66 ± 0.94 [a]	106.41 ± 0.25 [a]
	NaA@MAN-LPs	99.33 ± 2.62 [a]	100.57 ± 0.47 [a]	104.74 ± 0.58 [a]	105.93 ± 1.18 [a]
	NaA@LPs	102.26 ± 0.70 [a]	103.33 ± 0.98 [a]	105.81 ± 1.08 [a]	106.15 ± 0.93 [a]
	SC/MAN-LPs	101.57 ± 0.45 [a]	103.27 ± 2.26 [a]	104.75 ± 1.17 [a]	106.89 ± 0.37 [a]
PDI	NaA@SC/MAN-LPs	0.22 ± 0.00	0.21 ± 0.01	0.23 ± 0.01	0.22 ± 0.01
	NaA@SC-LPs	0.21 ± 0.00	0.22 ± 0.01	0.23 ± 0.01	0.24 ± 0.01
	NaA@MAN-LPs	0.23 ± 0.01	0.22 ± 0.01	0.22 ± 0.01	0.23 ± 0.02
	NaA@LPs	0.21 ± 0.00	0.21 ± 0.00	0.22 ± 0.01	0.23 ± 0.01
	SC/MAN-LPs	0.22 ± 0.01	0.21 ± 0.00	0.23 ± 0.01	0.23 ± 0.01
Zeta potential	NaA@SC/MAN-LPs	+ 31.80 ± 2.42	+ 30.20 ± 2.11	+ 30.60 ± 2.43	+ 31.26 ± 1.58
	NaA@SC-LPs	+ 32.30 ± 1.74	+ 32.90 ± 2.87	+ 30.20 ± 2.71	+ 31.96 ± 4.91
	NaA@MAN-LPs	+ 28.56 ± 2.58	+ 31.20 ± 1.75	+ 32.30 ± 1.74	+ 31.10 ± 2.1
	NaA@LPs	+ 28.05 ± 2.83	+ 29.29 ± 2.74	+ 30.10 ± 2.24	+ 33.20 ± 2.03
	SC/MAN-LPs	+ 27.72 ± 1.95	+ 30.10 ± 2.07	+ 31.62 ± 2.31	+ 32.80 ± 2.10
